# Genome-wide analysis of lipolytic enzymes and characterization of a high-tolerant carboxylesterase from *Sorangium cellulosum*

**DOI:** 10.3389/fmicb.2023.1304233

**Published:** 2023-12-04

**Authors:** Shu-Fei Yuan, Xin-Jing Yue, Wei-Feng Hu, Ye Wang, Yue-Zhong Li

**Affiliations:** State Key Laboratory of Microbial Technology, Institute of Microbial Technology, Shandong University, Qingdao, China

**Keywords:** lipolytic enzymes, family VIII carboxylesterase, *β*-lactamase, *Sorangium cellulosum*, myxobacteria

## Abstract

Microorganisms are important sources of lipolytic enzymes with characteristics for wide promising usages in the specific industrial biotechnology. The cellulolytic myxobacterium *Sorangium cellulosum* is rich of lipolytic enzymes in the genome, but little has been investigated. Here, we discerned 406 potential lipolytic enzymes in 13 sequenced *S. cellulosum* genomes. These lipolytic enzymes belonged to 12 families, and most are novel with low identities (14–37%) to those reported. We characterized a new carboxylesterase, LipB, from the alkaline-adaptive So0157-2. This enzyme, belonging to family VIII, hydrolyzed glyceryl tributyrate and *p*-nitrophenyl esters with short chain fatty acids (≤C12), and exhibited the highest activity against *p*-nitrophenyl butyrate. It retained over 50% of the activities in a broad temperature range (from 20°C to 60°C), alkaline conditions (pH 8.0–9.5), and the enzymatic activity was stable with methanol, ethanol and isopropanol, and stimulated significantly in the presence of 5 mM Ni^2+^. LipB also exhibited *β*-lactamase activity on nitrocefin, but not ampicillin, cefotaxime and imipenem. The bioinformatic analysis and specific enzymatic characteristics indicate that *S. cellulosum* is a promising resource to explore lipolytic enzymes for industrial adaptations.

## 1 Introduction

Lipolytic enzymes represent a group of proteins catalyzing the hydrolysis and formation of ester bonds of a structurally diverse array of compounds with no requirement for cofactors (Bornscheuer, 2002). Lipolytic enzymes can be employed for the synthesis of structurally diverse polymeric materials by catalyzing free combinations of diester and diol monomers (Kim and Dordick, 2001; Ning et al., 2022), forming chiral and enantioselective intermediates in the production of agrochemicals, flavoring compounds and pharmaceuticals (Tanaka et al., 2002; Athawale et al., 2003). Lipolytic enzymes are also used to degrade environmental toxic pesticides like pyrethroids, carbamate and organophosphate in an effective and green manner (Diegelmann et al., 2015; Sirajuddin et al., 2020). Significantly, lipolytic enzymes with high-tolerance characteristics, like thermophilic, cold-adaptive, alkaline, salt-tolerant, or stable in organic solvents, could bring higher yields and fewer by-products in the production of foods, detergents, fragrances and pharmaceuticals than those under mesophilic conditions (Priyanka et al., 2019; Al-Ghanayem and Joseph, 2020; Johan et al., 2021). With the increasing requirement of lipolytic enzymes for industrial biocatalysis, discovering novel lipolytic enzymes or remolding enzymes have attracted a lot of interests. Microbial lipolytic enzymes are widely used in industrial processes because of their potential broad substrate specificity, high region- and stereo-selectivity, and remarkable stability in organic solvents (Jaeger and Eggert, 2002; Panda and Gowrishankar, 2005). Exploring microbial genomic resources provides opportunities for deep excavation of novel lipolytic enzymes (Johan et al., 2021).

The lipolytic enzymes include two types, carboxylesterases (EC 3.1.1.1), hydrolyzing small water-soluble esters or triglycerides with fatty acids shorter than C6, and lipases (EC 3.1.1.3), which hydrolyze triglycerides composed of long-chain fatty acids. Both the carboxylesterase and the lipase belong to the alpha/beta-hydrolase superfamily and are characterized by having a catalytic triad composed of Ser, His and Asp (or Glu) residues and a conserved G-x-S-x-G, G-D-S-L or S-x-x-K motif around the nucleophilic serine at the active site (Holmquist, 2000; Bornscheuer, 2002). The classification system of bacterial lipolytic enzymes was first proposed from 53 enzymatic proteins by Arpigny & Jaeger, and included 8 families defined by the biochemical properties and sequence identities (Arpigny and Jaeger, 1999). With the discovery of more lipolytic enzymes, the bacterial lipolytic enzymes have been expanded to 19 families based on the phylogenetic criterium, conserved motifs and biological characteristics (Kovacic et al., 2018; Johan et al., 2021). Lipases are grouped in family I, including eight subfamilies, while carboxylesterases are reported in the rest of 18 families. Among these lipolytic enzyme families, family VIII carboxylesterases are unique for displaying both esterase and *β*-lactamase activities (Biver and Vandenbol, 2013; Mokoena et al., 2013; Jeon et al., 2016; Kwon et al., 2019), making them promising in the synthesis and modification of *β*-lactam antibiotics (Mokoena et al., 2013). The active serine residues of family VIII carboxylesterases are in the S-x-x-K motif, instead of the typical G-x-S-x-G pentapeptide, forming the catalytic triad with lysine and the other conserved tyrosine in the Y-x-x motif, the same as that of *β*-lactamases (Petersen et al., 2001; Cha et al., 2013).

The cellulolytic myxobacterium *Sorangium cellulosum* is not only extremely attractive in drug screening (Bollag et al., 1995; Gerth et al., 2003), but also exhibits extensive degradation abilities on a wide range of macromolecules, such as lipids and polysaccharides. In recent years, some novel glycoside hydrolases have been reported from this cellulolytic myxobacterium (Wang et al., 2012; Li et al., 2022), but little attention has been paid on lipolytic enzymes. *S. cellulosum* genomes have many ORFs (open reading frames) predicted to encode various hydrolytic enzymes (Schneiker et al., 2007; Han et al., 2013), and four lipolytic enzymes have been characterized (Cheng et al., 2011; Wu et al., 2012; Udatha et al., 2015), including the cold-adapted lipase LipA previously reported in the So0157-2 strain. Studying lipolytic enzymes with promiscuous activities will be helpful for our understanding of the cellulolytic myxobacteria and potential applications of the diverse enzyme resources. In this study, we identified the lipases and carboxylesterases from 13 available sequenced *S. cellulosum* genomes and characterized a novel family VIII carboxylesterase LipB, which was alkali-tolerant, feasible to a wide range of temperature, and especially stimulated by specific alcohols, suggesting potentials in industrial processing associated with alcohols or detergents production. Diverse lipases and carboxylesterases with potential adverse-tolerances from *S. cellulosum* genomes will conduce for the lipolytic enzyme applications in industrial production.

## 2 Materials and methods

### 2.1 Strains, plasmids, culture media and chemicals

Strains and plasmids used in this study are listed in Supplementary Table S1. *Escherichia coli* strains DH5α and BL21 (DE3) were used to clone plasmids and express the recombinant protein. *E. coli* strains were grown in Luria-Bertani (LB) broth at 37°C. *Myxococcus xanthus* strains were grown at 30°C in CYE medium [10 g/L casitone, 5 g/L yeast extract, 10 mM 3-(N-morpholino) propanesulfonic acid (MOPS) and 4 mM MgSO4, pH 7.6]. The media were supplemented with 40 μg/mL kanamycin, 30 μg/mL apramycin, or 10 μg/mL tetracycline if required. We employed the plasmids pET-28a and pET-29b as the expression vectors, while pBJ113 and pSWU30 were as the knock-out plasmid and the overexpression plasmid, respectively. Primers used in constructing plasmids are listed in Supplementary Table S2.

The substrates *p*-nitrophenyl (*p-*NP) acetate (C2), butyrate (C4), hexanoate (C6), octanoate (C8), decanoate (C10), laurate (C12), and *β*-lactam antibiotics of ampicillin, nitrocefin, cefotaxime, imipenem were purchased from Aladdin (Shanghai, China). Other chemicals used in this study were analytical grade unless otherwise specified.

### 2.2 Bioinformatics analysis of lipolytic enzymes in *Sorangium cellulosum* genomes

Lipolytic enzymes were identified from 13 *S. cellulosum* genomes by PSI-BLAST searches using representative enzymes of the 19 families as queries (num_interactions = 3, E-value cut-off = 10^−5^). The protein sequences were obtained from GenBank assembly of *S. cellulosum* genomes (Supplementary Table S3). The identified proteins were further filtered by the analysis of characteristic conserved motifs with FIMO^1^ and the reserved lipolytic enzymes were classified based on sequence identities with query sequences. The information of query sequences and consensus motifs of each lipolytic enzyme family is listed in Supplementary Table S4.

The sequence similarity network of 406 predicted *S. cellulosum* lipolytic enzymes was constructed with 171 studied lipolytic enzymes by EFI-EST (Oberg et al., 2023), the E-value for BLAST was set to 5 and the alignment score threshold was set at 10. Deductive amino acid sequences of the family VIII carboxylesterases were further aligned by MAFFT online version^2^ and embellished with ESPript (Robert and Gouet, 2014). The phylogenetic tree was constructed using the maximum likelihood method in IQ-TREE 2 (Minh et al., 2020) and modified by iTOL (Letunic and Bork, 2021).

### 2.3 Three-dimensional structure and docking analysis of LipB

To model the three-dimensional (3D) structure of the LipB protein, we submitted the amino acid sequence to I-TASSER online program based on a threading approach (Yang and Zhang, 2015) and visualized by PyMOL (Lilkova, 2015). AlphaFold2 (Cramer, 2021) was also applied to build the 3D structure of LipB, and models with predicted local-distance difference test (pLDDT) value of major sites above 70 were considered credible (Jumper et al., 2021). The accuracy of predicted structural models was assessed by SAVES v6.0.^3^ For molecular docking by AutoDock Vina (Trott and Olson, 2010; Eberhardt et al., 2021), the structure of LipB predicted by AlphaFold2 was employed as the receptor protein, and ligand molecules were downloaded in the mol2 format from the PubChem database.^4^ The docking results were visualized using PyMOL.

### 2.4 Expression and purification of recombinant LipB

Codon-optimized *lipB* sequence (Supplementary Table S5) was synthesized by GENEWIZ (Suzhou, China), amplified with the *lipB* F1/R1 and *lipB* F2/R2 primer pairs and cloned into the expression vectors pET-28a and pET-29b by homologous recombination with ClonExpress® MultiS One Step Cloning Kit (Vazyme, China) to generate recombinant plasmids pET-28a-*lipB* and pET-29b-*lipB*. For expression of the LipB protein, *E. coli* BL21 (DE3) harboring the recombinant plasmid was grown in 50 mL LB medium with 40 μg/mL kanamycin at 37°C to 0.6 of the OD600 value. Then isopropyl-*β*-D-thiogalactoside (IPTG) was added to the culture at a final concentration of 1 mM for additional 6 h incubation at 37°C or 0.1 mM for additional 22 h incubation at 16°C. The cells were collected by centrifugation and resuspended in Lysis buffer (25 mM Tris, 200 mM NaCl, 10% glycerin, pH 8.0), then crushed with an ultrasonic cell disruptor and the cellular supernatant was obtained by centrifugation at 12000 × g and 4°C for 30 min. The expression of the LipB protein was examined by SDS-PAGE.

To prepare the recombinant protein (LipB tagged with maltose-binding protein, MBP-LipB), *E. coli* BL21 (DE3) cells harboring the recombinant vector pET29b-*lipB* were cultured in 3 L of LB broth, and induced with 0.1 mM IPTG incubated at 16°C for 22 h. The supernatant was incubated with amylose affinity column (GE Healthcare, America), which was pre-equilibrated with Lysis buffer, and then eluted with the elution buffer containing 10 mM maltose. The soluble MBP-LipB protein was further purified using gel permeation chromatography to remove non-targeting proteins and finally resuspended in Lysis buffer.

### 2.5 Esterase activity assay of MBP-LipB

To assay the crude enzymatic activity, the *E. coli* BL21 (DE3) cells harboring the recombinant vector pET29b-*lipB* without and with induction of 0.1 mM IPTG at 16°C for 22 h were, respectively, harvested and resuspended with fresh LB broth at the concentration of 10 OD/mL, subsequently inoculated on the plate with glyceryl tributyrate, incubated overnight and observed by Stereo Microscope (Nikon, Japan).

The standard assay for esterase activity was carried out using spectrophotometric method with the reaction mixture containing 1 mM *p*-NP esters, 1 μL (0.67 μg) of purified MBP-LipB and 1% acetonitrile in a total volume of 1 mL of 50 mM Tris–HCl buffer (pH 8.0) (Petersen et al., 2001; Gupta et al., 2002). The reaction mixture was incubated for 10 min and terminated by the addition of 20 μL of 10% SDS. The enzymatic activity was measured by monitoring the changes of absorbance at 405 nm. All measurements were performed in triplicate.

Substrate specificity was detected by using *p*-NP esters with different length of aliphatic side chains, including *p*-NP acetate (C2), *p*-NP butyrate (C4), *p*-NP hexanoate (C6), *p*-NP octanoate (C8), *p*-NP decanoate (C10), and *p*-NP laurate (C12).

The optimal pH value was determined with *p*-NP butyrate as the substrate in the pH range from 3.0 to 10.0. The following buffers (50 mM) with different pH values were used: citrate buffer (pH 3.0–5.0), sodium phosphate buffer (pH 5.0–7.0), Tris–HCl (pH 7.0–9.0) and sodium bicarbonate–NaOH buffer (pH 9.0–10.0).

Similarly, the optimal temperature was determined at the temperatures ranging from 20°C to 70°C. The thermostability was determined by incubating the reaction mixtures at 35°C, 45°C, 55°C for different times until up to 1 h and the residual activity was measured.

Effects of metal ions (MnCl_2_, MgCl_2,_ CaCl_2_, CuCl_2_, CoCl_2_, ZnCl_2_ and NiCl_2_) or organic solvents (methanol, ethanol, acetone, trichloromethane, acetonitrile and isopropanol) on LipB esterase activity were detected by incubating the reaction mixture, respectively, with the metal ions or organic solvents under the reaction condition mentioned above for 1 h, and the residual activity subsequently tested. The concentration of each of the metal ions was 5 mM, and the final concentrations of the organic solvents were 5%, 10% or 15%. The enzymatic activity of the protein without additives was defined as 100%.

### 2.6 *β*-lactamase activity assay of MBP-LipB

The *β*-lactamase activity of LipB was determined by using nitrocefin as the substrate with the method (O'Callaghan et al., 1972) with small modifications. Briefly, the reaction mixture containing 1 mM nitrocefin, 1 μL purified enzyme (0.67 μg), 500 μL of 200 mM Tris–HCl buffer (pH 7.0) and distillation-distillation water (ddH_2_O) in a total 1 mL volume was incubated at 30°C and measured with the spectrophotometric method at 482 nm every 1 h. To exclude the influence of the MBP-tag, we used the MBP protein in the *β*-lactamase activity assay with nitrocefin as the substrate.

Ampicillin, cefotaxime or imipenem was also used as substrate to detect the *β*-lactamase activity of MBP-LipB using the same reaction mixture without nitrocefin and then incubated at 30°C for 24 h. The concentrations of residual substrates and reaction products were determined by High Performance Liquid Chromatography (HPLC) equipped with a C18 reverse-phase column (Thermo Fisher Scientific, Boston, USA). The elution condition was a constant concentration gradient of phosphate buffer and acetonitrile (HPLC grade) at a flow rate of 0.5 mL/min for 20 min to detect at 230 nm for ampicillin, at a flow rate of 0.8 mL/min for 20 min to detect at 254 nm for cefotaxime, and at a flow rate of 1 mL/min for 15 min to detect at 295 nm for imipenem. The reaction metabolites were identified by comparing the retention time and the UV visible spectra with the negative control using ddH_2_O to replace the enzyme.

The optimal temperature and pH for LipB *β*-lactamase activity were employed. Because nitrocefin was unstable under the thermal (≥55°C) or alkaline (pH ≥8.0) conditions, the detection was conducted at the temperature range from 20°C to 50°C or in the pH range from 3.0 to 7.5.

### 2.7 Epothilone hydrolase activity assay of MBP-LipB

To analyze the hydrolase activity of MBP-LipB against epothilones, 0.5 mM epothilone A or epothilone B and 2 μg of purified enzyme were added into 50 mM Tris–HCL (pH 9.0) at a final volume of 100 μL and incubated at 30°C for 24 h. An equal volume of ethyl acetate was added to finish the reaction, the mixture was evaporated under reduced pressure and dissolved in 50 μL methanol. The remained epothilone A or epothilone B was determined by HPLC. The elution condition was a programmed concentration gradient of 60% methanol (HPLC grade) and 40% ddH_2_O (HPLC grade) at a flow rate of 1 mL/min for 25 min to detect at 249 nm.

Primers *lipB*-up F and *lipB*-up R, *lipB*-down F and *lipB*-down R were used to amplify the up and down homologous arms of *lipB* from *S. cellulosum* So0157-2 genome, respectively. The arms were linked to pBJ113 to obtain knockout plasmid pBJ-*lipB*. The *lipB* gene was amplified with *lipB* F3 and *lipB* R3 primers, digested with KpnI and EcoRI and then cloned into pSWU30-pilA resulting in the overexpression plasmid pSWU30-pilA-*lipB*. The pBJ-*lipB* and pSWU30-pilA-*lipB* were introduced into the epothilone-producing strain ZE9 (Zhu et al., 2015) by electroporation and the positive mutant strains ZE9∆*lipB* and ZE9 + *lipB* were screened as previously reported (Yue et al., 2017). ZE9 and mutants were cultivated overnight in 50 mL of CYE medium, then inoculated at a ratio of 0.04 OD/mL into 50 mL medium containing 2% of the XAD-16 resin and fermented at 30°C for 7 days. The resin was harvested with strainer and extracted with 3 mL methanol by shaking overnight at room temperature (Gong et al., 2007). The supernatant was examined by HPLC. The yield of epothilones was quantified from the peak area in the UV chromatogram, by reference against a calibration standard.

## 3 Results

### 3.1 Identification of lipolytic enzymes in *Sorangium cellulosum*

**Figure 1:**
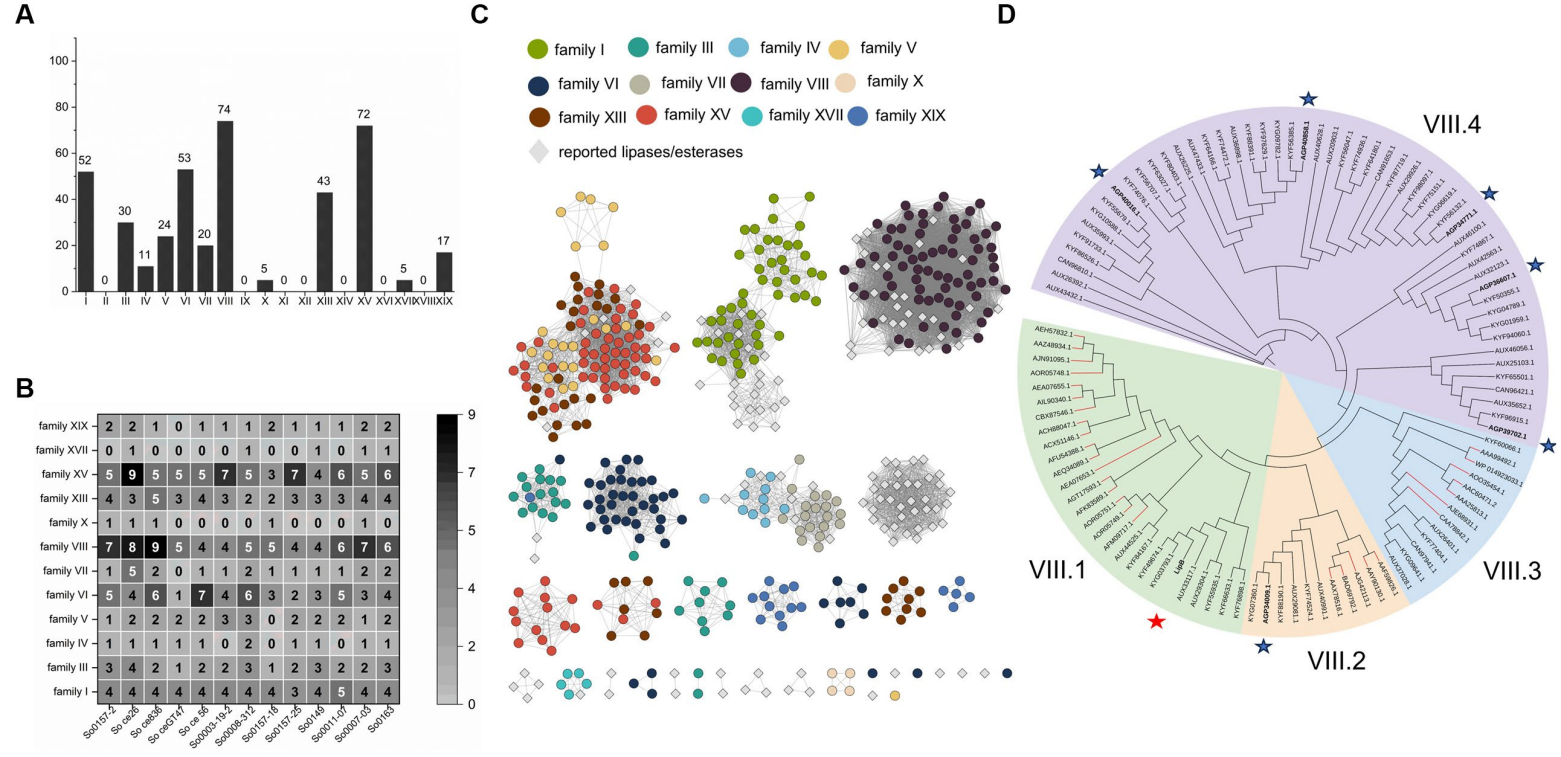
Identification and amino acid sequences analysis of lipolytic enzymes from *S. cellulosum*. **(A)** The amount of lipolytic enzymes of each gene family from *S. cellulosum* was plotted with the histogram and labeled on top of each bar. **(B)** The number of each lipolytic enzyme family in various *S. cellulosum* genomes was counted with the heat map. The number larger than or equal to 5 was marked in white, otherwise in black. **(C)** The sequence similarity network constructed by EFI-EST. Dots represent enzymes identified from *S. cellulosum*, of them different enzyme families were exhibited in various colors. Reported enzymes were displayed with gray diamonds. **(D)** The phylogenetic tree of family VIII carboxylesterases established by IQ-TREE. These enzymes were divided into four groups. There were 29 reported family VIII esters, of which the branches were painted red. Seven enzymes from *S. cellulosum* So0157-2 were bold and highlighted with asterisks, and the LipB was highlighted with red asterisk.

We searched the 13 available *S. cellulosum* genomes (Supplementary Table S3) by PSI-BLAST with 19 representatives from those identified lipolytic enzymes of different families as query sequences, and discerned 1,084 non-redundant lipolytic enzymes, which belonged to 14 families (Supplementary Table S6). These putative enzymes were filtered with FIMO to determine the existence of the typical motifs conserved in lipolytic enzyme families (Johan et al., 2021). After removing the sequences without the conserved motifs we obtained 406 lipolytic enzymes (Supplementary Table S7). Notably, because the conserved motifs in the families III, VI, XV and XIX, or in the families IV and VII, were closely similar, 61 of the 406 proteins appeared in different families, which were determined of their ascription by the BLASTP similarity values (Supplementary Table S8). Finally, these *S. cellulosum* lipolytic enzymes were classified into 12 families (Figure 1A).

The *S. cellulosum* genomes each contained multiple genes (22–44) encoding lipolytic enzymes (Supplementary Table S9), with varied compositions in different families (Figure 1B). According to the sequence similarity network analysis, the lipolytic enzymes belonging to families I, IV, VII, VIII, and XVII showed high similarities, but many others (up to 60% of the 406 enzymes) exhibited low similarities with those reported representatives (Figure 1C), showing rich novel lipolytic enzymes in *S. cellulosum*.

The family VIII carboxylesterases were the most abundant lipolytic enzymes occurring in *S. cellulosum*. These 74 predicted family VIII carboxylesterases, together with 29 reported ones, could be divided into four groups (Figure 1D). To understand lipolytic enzymes in *S. cellulosum*, we further investigate the sequence and functional characteristics of LipB (AKI82204.1) of the family VIII carboxylesterases in *S. cellulosum* So0157-2, an alkaline epothilone-producing strain with the known largest *S. cellulosum* genome (Han et al., 2013). One more reason for the choice of LipB is that the *lipB* gene is adjacent to the biosynthetic gene cluster of epothilones, and the LipB protein was once predicted to be an esterase responsible for the hydrolysis of epothilones to prevent self-toxicity (Gerth et al., 2002; Zhao et al., 2010; Li et al., 2017).

### 3.2 Sequence alignment and three-dimensional structure of LipB

**Figure 2:**
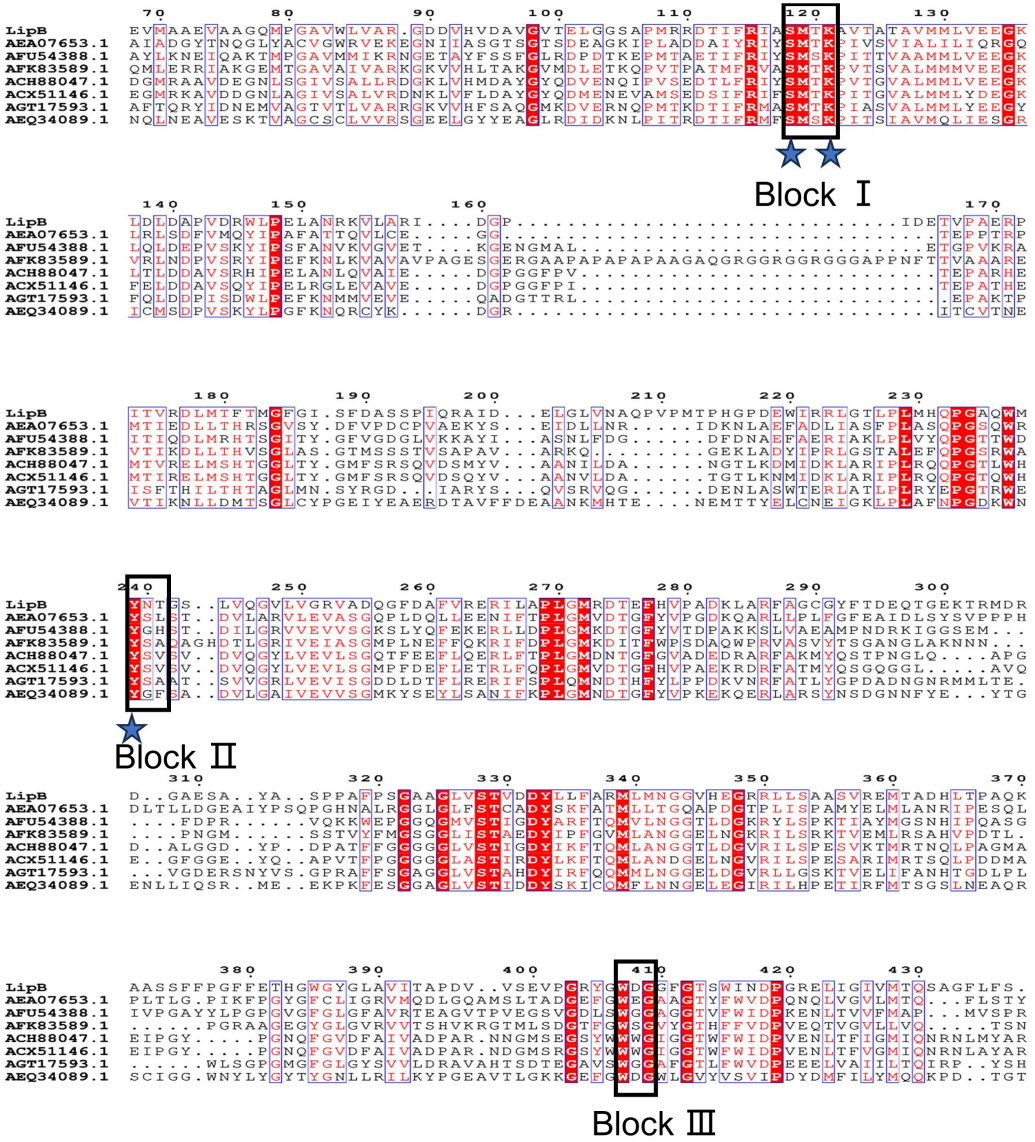
Multiple amino acid sequences alignment of LipB and reported homologs. Identical residues are indicated by white text on a red background and similar residues are shown in red text on a white background. Groups of residues with a global similarity score above 0.7 are framed in blue. The pivotal conserved motifs S-x-x-K, Y-x-x, and W-x-G are marked as Block I, Block II and Block III, respectively. The putative catalytic triad (Ser118, Lys121 and Tyr239) is indicated by blue asterisks.

So0157-2 contained 34 lipolytic enzymes, and 7 of them belonged to the family VIII carboxylesterases. The *lipB* gene encodes a protein containing 454 amino acid residues with the predicted molecular weight of 48.4 kDa. Multiple amino acid sequence alignment revealed that LipB contained the conserved S-x-x-K motif (at the position of residues of 118–121) and Y-x-x motif (239–241), which are commonly observed in class C *β*-lactamases, penicillin binding proteins and family VIII carboxylesterases (Figure 2). Besides, the W-x-G motif, conserved in the oxyanion hole of family VIII carboxylesterases (Nan et al., 2019; Park et al., 2020), was also observed in the C-terminal region of LipB (Trp^407^-Asp^408^-Gly^409^). The sequence characteristics suggested that LipB was a dual-functional enzyme with the class C *β*-lactamase and the family VIII carboxylesterase activities.

**Figure 3:**
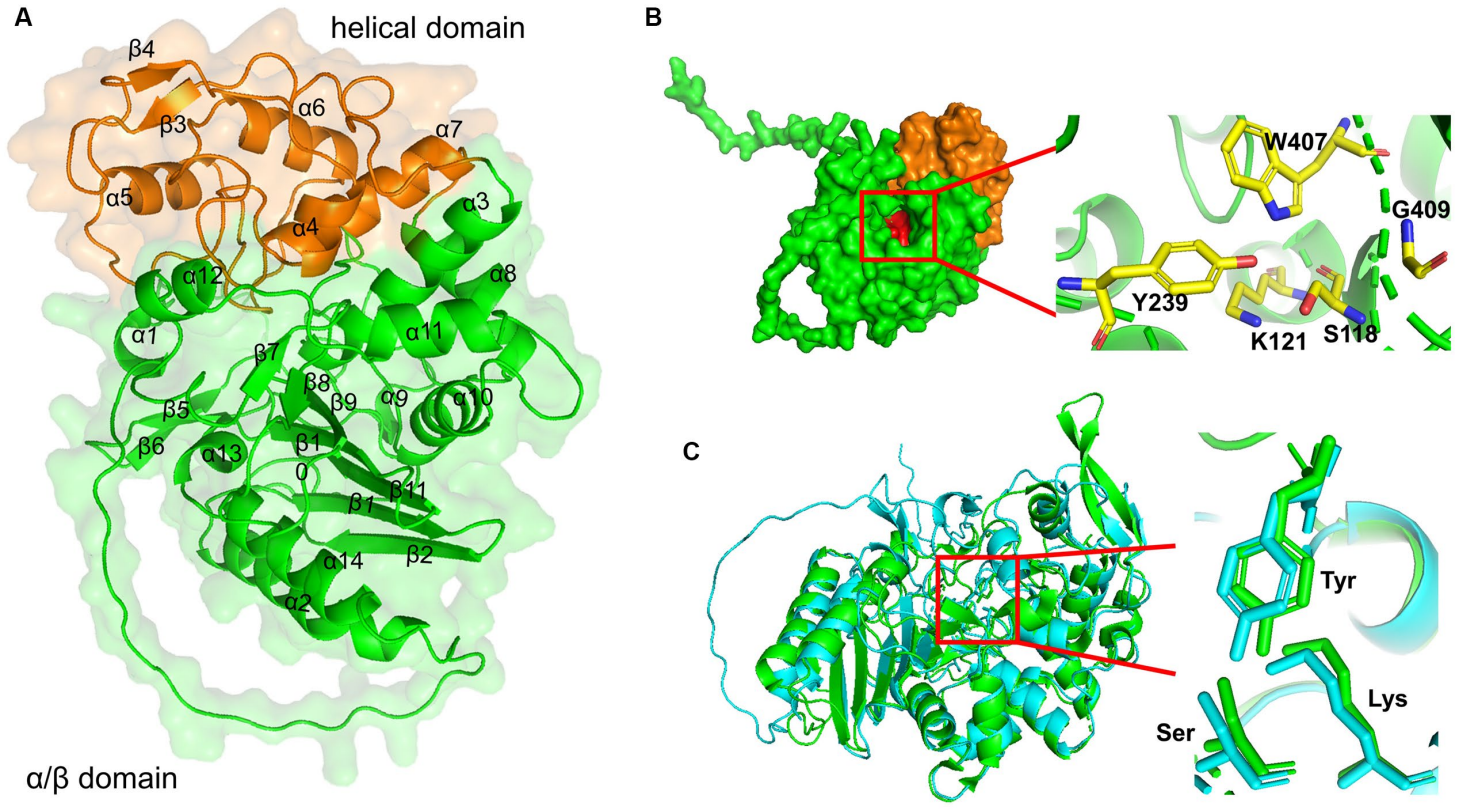
3D structure modeling of LipB. **(A)** A ribbon diagram of LipB was shown with the transparent surface structures. The secondary structures were labeled in black. The protein was divided into two domains: an alpha/beta-domain (residues 1–135 and residues 258–454) shown in green and a helical domain (residues 136–257) shown in orange. **(B)** Three conserved motifs were shown in red by the surface drawing. And the key residues (S118 and K121 in S-x-x-K motif, Y239 in Y-x-x motif, W407 and G409 in W-x-G motif) were zoomed clearly and shown as sticks in the ribbon diagram of LipB on the right. **(C)** Alignment of 3D structures of EstU1 and LipB. EstU1 (PDB code: 4IVI) and LipB were colored in green and cyan, respectively. The key residues related to *β*-lactam hydrolytic activity in catalytic triad were shown in sticks and zoomed at the right.

We constructed the 3D structure of LipB protein using the I-TASSER online program, and revealed that LipB was structurally close to some carboxylesterases (PDB IDs: 4IVI, 1CI8, and 3ZYT) and several penicillin binding proteins (PDB IDs: 4P6B, 5GKV, and 2QMI) (Supplementary Table S10). The optimal structures predicted by I-TASSER and AphaFold2 (model 1 and rank_1) were aligned and matched well with each other (Supplementary Figure S1A), and residues, 65.1% of model 1 and 85.1% of rank_1, revealed by ramachandran plot analysis, were in the most favored regions (Supplementary Figure S1B). According to Verify3D, 68.9% of residues in model 1 have scored ≥0.2 in the 3D-1D profile, while the residues scored ≥0.2 in the rank_1 were 70.9% (Supplementary Figure S1C). Therefore, the rank_1 model estimated by AlphaFold2 (pLDDT:87.3, pTM:0.857) was adopted as the supposed 3D structure of LipB (Figure 3A).

Similar to the class C *β*-lactamase and family VIII carboxylesterases (Wagner et al., 2002), LipB was composed of two domains, a small helical domain (residues 136–257, painted orange in Figure 3A) and an alpha/beta-domain (residues 1–135 and residues 258–454, painted green). The helical domain consisted of four alpha-helices and a short two-stranded antiparallel beta-sheet. The alpha/beta-domain had five long antiparallel beta-sheets, two pairs of short two-stranded antiparallel beta-sheet and 10 alpha-helices (7 on one side and 3 on the other). These two domains shaped a catalytic active pocket, where three conserved motifs (S-x-x-K, Y-x-x, W-x-G, painted red in surface) were precisely fit (Figure 3B). As shown in Figure 3C, a structure superimposition (root-mean-square deviation (RMSD) of 1.1 Å) was observed between LipB and EstU1 (PDB ID: 4IVI), a characterized family VIII carboxylesterase with the *β*-lactamase activity on the first-generation cephalosporins, and the key active sites essential for the *β*-lactam hydrolytic activity overlapped well in LipB (Ser^118^, Lys^121^ and Tyr^239^) and EstU1 (Ser^100^, Lys^103^ and Tyr^218^). The above bioinformatics analysis further suggested that LipB might display both esterase and *β*-lactamase activities.

### 3.3 Expression and purification of recombinant MBP-LipB

To investigate the biological activity of this enzyme, we expressed the recombinant LipB in *E. coli*. The codon optimized *lipB* gene was cloned in different expression vectors and transformed into *E. coli* BL21 (DE3). With the expression plasmid pET28a-*lipB*, the His-LipB recombinant proteins were expressed in an insoluble form, even after optimization of the induction conditions (Supplementary Figure S2A, the band of His-LipB was marked with red arrows). When labeled with the MBP-tag at the N-terminal of LipB, MBP-LipB was solubly expressed in cells harboring pET-29b-*lipB*; more recombinant proteins were obtained with the induction with 0.1 mM IPTG at 16°C for 22 h than that induced by 1 mM IPTG at 37°C for 6 h (Supplementary Figure S2B). The recombinant proteins were purified with amylose affinity chromatography and gel permeation chromatography. Notably, if the MBP-tag was truncated from MBP-LipB, the LipB protein became insoluble. Thus, the recombinant MBP-LipB protein was employed in the following assays.

### 3.4 Biochemical characterization of LipB as an esterase

**Figure 4:**
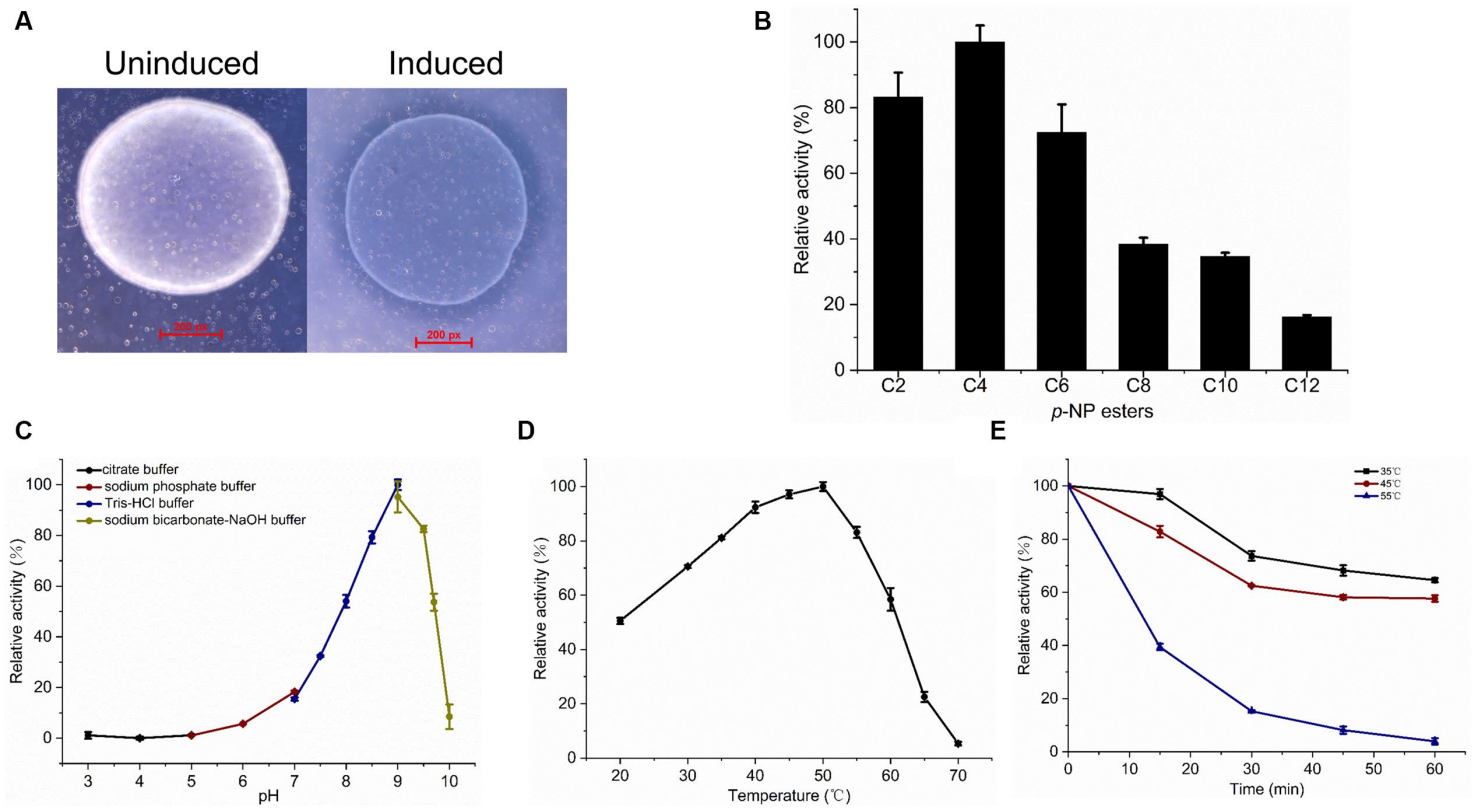
Esterase activity assay and biochemical characterization of recombinant LipB. **(A)** Hydrolysis of glyceryl tributyrate by LipB. Transparent zone was observed around the induced colonies. **(B)** Relative activity of LipB at *p*-NP esters with different chain lengths of fatty acids. Substrates used were *p*-NP acetate (C2), *p*-NP butyrate (C4), *p*-NP hexanoate (C6), *p*-NP octanoate (C8), *p*-NP decanoate (C10) and *p*-NP laurate (C12). **(C)** Effect of pH on activity of LipB. Activities under various pH conditions were marked by black (pH 3.0–5.0), wine (pH 5.0–7.0), navy (pH 7.0–9.0), dark yellow (pH 9.0–10.0). **(D)** Effect of temperature on activity of LipB. **(E)** Thermostability of LipB. The enzyme was preincubated at different temperatures for different time quantum, and the residual activity was determined. The residual activities incubated at 35°C, 45°C and 55°C were marked by black, wine and navy, respectively.

To determine the esterase activity of LipB in *E. coli*, the IPTG-induced and uninduced cells harboring pET-29b-*lipB* were inoculated on the plates supplemented with glyceryl tributyrate. After overnight incubation, an obvious transparent zone was observed around the induced colonies, but not the uninduced, indicating that the induced LipB could hydrolyze the glyceryl tributyrate (Figure 4A). Subsequently, we purified the MBP-LipB proteins and assayed the esterase activity with the substrates *p*-NP esters with various chain lengths of fatty acids (from C2 to C12). As shown in Figure 4B, LipB efficiently hydrolyzed *p*-NP esters with short chain fatty acids and exhibited the highest activity toward *p*-NP butyrate (C4). Defining the hydrolytic activity toward *p*-NP butyrate as 100%, LipB maintained more than 70% of activity against *p*-NP esters with acetate (C2) or hexanoate (C6). When *p*-NP esters were with longer chain lengths (C8 and C10), the esterase activities were less than 40%, and dropped to 20% with the *p*-NP laurate (C12) as a substrate. The absorbance of *p*-NP esters incubation showed no activity with the MBP protein, which excluded the influence of MBP-tag in esterase activity of LipB (Supplementary Figure S3A). In addition, the structure modeling also showed that the MBP and LipB fragments formed two separate parts with no significant interactions (Supplementary Figure S3B). Notably, the activities of LipB were some different from that of LipA of the same strain, which belongs to family XV and exhibited the highest activity toward *p*-NP acetate (C2) under various pH and temperature conditions (Cheng et al., 2011).

Effect of pH on the esterase activity of LipB was measured in a pH range from 3 to 10 with *p*-NP butyrate as the substrate. As shown in Figure 4C, the LipB protein exhibited high activity under an alkaline condition (pH 8.0–9.5) and the maximum activity appeared at pH 9.0, which was corresponding to the natural growing environment of *S. cellulosum* So0157-2 (Han et al., 2013). Under the conditions with pH value lower than 7.0 or higher than 10.0, LipB lost more than 80% of activity.

LipB retained high activity in a broad temperature range (from 20°C to 60°C) and exhibited the maximum activity at 50°C. When the temperature increased to 70°C, the enzyme lost its activity almost completely (Figure 4D). Thus, unlike the cold-adapted lipase LipA identified previously, LipB worked as a thermophilic family VIII carboxylesterase. As to the temperature stability, LipB retained about 60% of activity after an incubation at 35°C or 45°C for 60 min. However, 30-min incubation at 55°C diminished more than 80% of the enzymatic activity (Figure 4E).

The activities of LipB in the presence of different metal ions are shown in Table 1. In general, lipolytic enzymes do not need cofactors in the hydrolyzation of the ester-bond. Nevertheless, it has been reported that activities of lipases and carboxylesterases are also enhanced by some divalent cations such as Ca^2+^, Zn^2+^, and Mg^2+^ (Choi et al., 2004; Gao et al., 2016; Araujo et al., 2020). As to LipB, addition of Ca^2+^, Cu^2+^ or Zn^2+^ reduced the activity approximately in half, whereas the presence of 5 mM of Mg^2+^, Co^2+^ or Ni^2+^ enhanced LipB esterase activity, and the highest increase to 142.8% was obtained by the addition of Ni^2+^. As suggested by Araujo et al. (2020), the activity of LipB might be strengthened by the promotion of these divalent cations on a rapid product release or cleaning the enzyme’s active sites.

**Table 1:** Effects of metal ions on the esterase activity of LipB.

Cations	Relative activity (%) ^a^
Control	100
MnCl_2_	92.6 ± 5.6
MgCl_2_	112.0 ± 1.3
CaCl_2_	66.3 ± 5.3
CuCl_2_	52.4 ± 2.3
CoCl_2_	117.6 ± 9.2
ZnCl_2_	50.0 ± 5.1
NiCl_2_	142.8 ± 1.3

^a^ The relative activities are given as a percentage of the activity in the absence of cations.

More interestingly, LipB exhibited superior tolerance to organic solvents. The activity of LipB was stimulated 2-fold, 2.7-fold and 1.6-fold by the presence of 15% of methanol, 10% of ethanol or 5% of isopropanol, respectively, but suppressed by acetone, trichloromethane and acetonitrile. 10% of trichloromethane or 15% of acetonitrile inactivated LipB completely (Table 2). The organic solvent stability is an important criterion for industrial esterases (Gorman and Dordick, 1992). The excellent organic solvent stability of LipB implied its potentials in industrial applications for biotransformation and bioremediation associated with organic solvents.

**Table 2:** Effects of organic solvents on the esterase activity of LipB.

Organic solvents	Relative activity (%) ^a^ at various solvent concentrations (%) of		
	5	10	15
Control	100	100	100
Methanol	248.6 ± 16.3	227.3 ± 5.6	203.8 ± 7.7
Ethanol	256.7 ± 17.6	272.4 ± 8.5	94.3 ± 5.3
Acetone	78.6 ± 4.7	49.0 ± 7.9	12.2 ± 4.3
Trichloromethane	7.4 ± 8.6	— ^b^	—
Isopropanol	156.0 ± 11.6	116.4 ± 3.1	—
Acetonitrile	54.9 ± 4.0	11.3 ± 3.1	—

^a^ The relative activities are given as a percentage of the activity in the absence of organic solvents.

^b^ — represents the inactive state of enzyme.

### 3.5 *β*-lactamase activity of LipB

**Figure 5:**
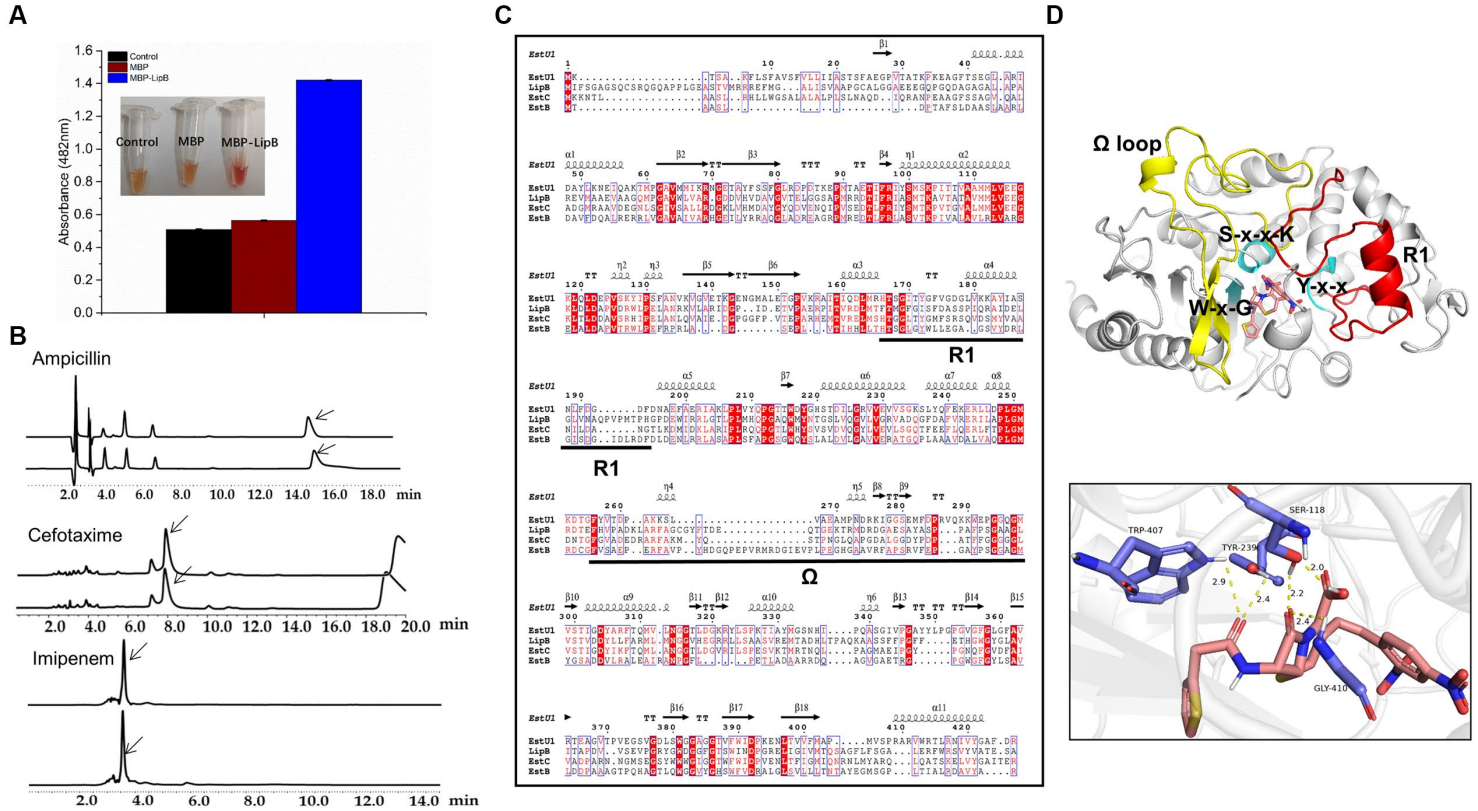
Activity of LipB toward *β*-lactam antibiotics. **(A)** Color change of nitrocefin-containing solution with MBP and MBP-LipB, using reaction mixture without proteins as a blank control. Specific data of absorbance at 482 nm is shown in the histogram form. **(B)** High performance liquid chromatography profiles for *β*-lactam antibiotics (upper) and *β*-lactam antibiotics incubated with LipB (nether). The substrate peaks of ampicillin, cefotaxime and imipenem are at 15.4 min, 7.9 min and 3.5 min, which were marked with arrows. **(C)** Sequence alignment among LipB, EstU1 (AFU54388.1), EstC (ACH88047.1) and EstB (AAF59826.1). The secondary structure assignment corresponds to EstU1. Ω represents the Ω loop and R1 represents R1 segment. **(D)** Molecule docking of LipB with the substrate nitrocefin and interactions of key sites. The predicted LipB/nitrocefin complex was shown at the image upper, Ω and R1 represent Ω-loop (yellow, residues 277–325) and R1 segment (red, residues 181–216), respectively. S-x-x-K motif, Y-x-x motif and W-x-G motif are marked by cyan. The potential hydrogen bond interactions of serine, tyrosine, tryptophan and glycine toward nitrocefin was displayed below.

According to the *β*-lactamase activity, family VIII carboxylesterases were classified into three types: having no *β*-lactamase activity, represented by EstB (Petersen et al., 2001); only active to nitrocefin, represented by EstC (Rashamuse et al., 2009); and having *β*-lactamase activities toward different *β*-lactam antibiotics including cephaloridine, cefazolin, cephalothin and nitrocefin, represented by EstU1 (Jeon et al., 2011). We used four *β*-lactam antibiotics as the substrates for the *β*-lactamase activity assay: ampicillin, nitrocefin, cefotaxime and imipenem (Supplementary Figure S4). As shown in Figure 5A, the absorbance of nitrocefin at 482 nm raised to 1.42 after incubation with MBP-LipB, and the color of solution changed from yellow to red which indicated that LipB could hydrolyze the amide bond of nitrocefin (Lee et al., 2005), and exhibited the maximum *β*-lactamase activity at 40°C or pH 7.0 (Supplementary Figure S5). The *β*-lactamase activities against ampicillin, cefotaxime and imipenem by LipB were also analyzed by HPLC, but no novel peaks derived from the substrate hydrolysis were observed (Figure 5B), illustrating that LipB had no *β*-lactamase activity toward these three antibiotics. Thus, LipB mimicked the *β*-lactamase activity of EstC, although the structure of LipB was closer to EstU1 (Figure 3C).

It was previously reported that appropriate length of the Ω-loop and the R1 segment (the connecting region between α6 and α8) in EstU1 played a critical role for its substrate hybridity to multiple *β*-lactam antibiotics, and the long Ω-loop might cover the R1 site and block the access to the catalytic triad (Cha et al., 2013). We aligned the amino acid sequences of LipB, EstU1, EstC and EstB, and found that the Ω-loop of LipB was 7 residues longer than that of EstU1, 4 residues longer than that of EstC, and 9 residues shorter than that of EstB (Figure 5C), which suggested that, compared with EstU1 and EstB, the intermediate length of Ω-loop might prompt LipB to form a conformation that allowed the moderate *β*-lactamase activity.

In addition, based on the docking model estimated by AutoDock, the nitrocefin was well located in the active pocket without blocking from the moderate Ω-loop of LipB. Serine in the S-x-x-K motif, tyrosine in the Y-x-x motif, and tryptophan in the W-x-G motif formed hydrogen bonds interaction with the ligand nitrocefin, and the carbonyl oxygen in the *β*-lactam ring of nitrocefin was located at the oxyanion hole derived from Ser118 and Gly410 (Figure 5D). While in the docking model of LipB/ampicillin, the carbonyl oxygen of the opened *β*-lactam ring of ampicillin was interacted with Ser118 and Tyr239 instead of the oxyanion hole (Supplementary Figure S6A), which may affect the occurrence of the second reaction of hydrolysis. For cefotaxime and imipenem, no key residue in active pocket was linked to the lactam ring of substrates in the docking models with LipB (Supplementary Figures S6B,C), explaining why they could not be hydrolyzed by LipB.

## 4 Discussion

Diverse microbial lipolytic enzymes exhibited versatile application potentials with their catalytic activities on various substrates under adverse conditions (Bornscheuer, 2002). *S. cellulosum* is an intriguing but unexploited resource for lipolytic enzymes screening. In the 13 sequenced *S. cellulosum*, we discerned hundreds of lipolytic enzymes belonging to 12 families. In addition to the LipA previously reported in *S. cellulosum*, the LipB also exhibited excellent properties potential for specific industrial processing. Hence deep exploration of *S. cellulosum* is promising to provide more novel candidate lipolytic enzymes for the various requirements of industrial biotechnology. Notably, although LipB was once predicted to be responsible for the hydrolysis of epothilones (Gerth et al., 2002; Zhao et al., 2010; Li et al., 2017), our *in vitro* and *in vivo* analyses indicated that the enzyme was unable to hydrolyze epothilones (Supplementary Figure S7).

The broad substrate spectrum and harsh environment tolerance prompt family VIII carboxylesterases to be potentially applied in pharmaceutical, organic synthesis and other industrial productions, but only dozens of enzymes of this family have been investigated so far. Bioinformatics analysis shows that there were normally many family VIII carboxylesterases in *S. cellulosum* genomes, which were deserved for further investigation. Exemplified in this study, we analyzed the sequence and functional characteristics of LipB, a family VIII carboxylesterase. LipB had esterase activity toward glyceryl tributyrate and *p*-NP esters with short length of aliphatic side chains and weak *β*-lactamase activity against nitrocefin. The enzyme was alkaline and exhibited excellent activities in a wide range of temperature. Moreover, LipB was well tolerant to organic solvents, and even stimulated by methanol, ethanol, and isopropanol, which might indicate potential application in specific industrial processing associated with alcohols solvents. Similarly, several family VIII carboxylesterases were reported to be stimulated by methanol (Rashamuse et al., 2009; Selvin et al., 2012; Ouyang et al., 2013; Lee et al., 2016). It was confirmed by Müller et al. that some methanol-stimulated esterase could catalyze the acylation of methanol and the acyl-enzyme intermediate would be rapidly disassociated to accelerate the release of *p*-nitrophenol and results in higher hydrolysis activities (Müller et al., 2021).

Family VIII carboxylesterases show different hydrolase activities against different type of *β*-lactam antibiotics. Although the key active sites (Ser, Lys and Tyr) essential for the *β*-lactamase activity overlapped well in the three-dimensional structures of LipB and EstU1, LipB catalyzes the hydrolysis of only nitrocefin, but not ampicillin, cefotaxime and imipenem. The spatial adaptation of LipB might be a more essential criterion for *β*-lactamase activity according to the results of sequence alignment and AutoDock.

## 5 Conclusion

In this study, we discerned a total of 406 lipolytic enzymes in 13 *S. cellulosum* genomes, and most of them exhibit low sequence similarity with those reported. We characterized a family VIII carboxylesterase LipB, alkaline, feasible to a wide range of temperature, and especially stimulated by organic solvents like methanol, ethanol and isopropanol. We propose that *S. cellulosum* strains are a treasury for digging more novel and promising industrial lipolytic enzymes.

## Data availability statement

The original contributions presented in the study are included in the article/Supplementary material, further inquiries can be directed to the corresponding authors.

## Author contributions

S-FY: Data curation, Investigation, Methodology, Software, Visualization, Writing – original draft, Writing – review & editing. X-JY: Funding acquisition, Project administration, Resources, Supervision, Writing – review & editing. W-FH: Methodology, Software, Writing – review & editing. YW: Methodology, Software, Writing – review & editing. Y-ZL: Funding acquisition, Project administration, Resources, Supervision, Validation, Writing – review & editing.

## Footnotes

^1^ https://meme-suite.org/meme/tools/fimo

^2^ https://mafft.cbrc.jp/alignment/server/

^3^ https://saves.mbi.ucla.edu/

^4^ https://pubchem.ncbi.nlm.nih.gov/
