# Supplementary material for: Genome-wide analysis of lipolytic enzymes and characterization of a high-tolerant carboxylesterase from Sorangium cellulosum
Source: Front Microbiol. 2023 Dec 4;14:1304233. doi: 10.3389/fmicb.2023.1304233 (PMC10725956; doi:10.3389/fmicb.2023.1304233)
Supplement: Supplementary file 1 [file Table_1.DOCX]

**Table S1.** Strains and plasmids used in this study.

| **Strain and plasmid** | **Relevant characteristics** | | **Source or reference** |
| --- | --- | --- | --- |
| Strain | |  |  |
| *E. coli* |  | |  |
| DH5α  BL21 (DE3) | F−, *supE44, ΔlacU169 (ϕ80lacZΔM15), hsdR17, recA1, endA1, gyrA96, thi‑1, relA1* | | Life Technologies  Life Technologies |
| *M. xanthus* |  | |  |
| ZE9 | A heterologous expressional host of epothilone, derived from *M. xanthus* DZ2 | | This lab |
| ZE9∆*lipB*  ZE9+*lipB* | ZE9 with deletion of *lipB* gene  ZE9 with integration of pSWU30-pilA-*lipB* | | This study  This study |
| Plasmid |  | |  |
| pET-28a | Expression vector | | Novagen |
| pET-28a-*lipB*  pET-29b  pET-29b-*lipB* | Ligating the *lipB* gene to the pET-28a  Expression vector  Ligating the *lipB* gene to the pET-29b | | This study  Novagen  This study |
| pBJ113 | Gene replacement vector with KG cassette; Kanamycin^r^ | | Z.M. Yang, Virginia Tech |
| pBJ-*lipB*  pSWU30  pSWU30-pilA  pSWU30-pilA-*lipB* | Ligating two homologous arms upstream and downstream of *lipB* gene to the pBJ113  Site specific integration vector with Mx8 attB integration site (Mx8); Tetracycline^r^  Ligating the promoter of *pilA* gene to the pSWU30  Ligating the *lipB* gene to the pSWU30-pilA | | This study  (Wu and Kaiser 1995)  This lab  This study |
