# Supplementary material for: Genome-wide analysis of lipolytic enzymes and characterization of a high-tolerant carboxylesterase from Sorangium cellulosum
Source: Front Microbiol. 2023 Dec 4;14:1304233. doi: 10.3389/fmicb.2023.1304233 (PMC10725956; doi:10.3389/fmicb.2023.1304233)
Supplement: Supplementary file 2 [file Table_2.DOCX]

**Table S2.** Primers used in construction of plasmids and mutant strains.

| **Primer name** | **Sequence (5'-3')** | **Restriction Site** | |  |
| --- | --- | --- | --- | --- |
| *lipB* F1 | | ACGGAGCTCGAATTCGGCGCTTTCCGTCGC | | None |
| *lipB* R1 | | ATGGGTCGCGGATCCATCTTCAGTGGCGCG | | None |
| pET-28a F | | GGATCCGCGACCCATTTGCT | | None |
| pET-28a R | | GAATTCGAGCTCCGTCGACAA | | None |
| *lipB* F2 | | TTGTCGACGGAGCTCGAATTGGCGCTTTCCGTCGCCAC | | None |
| *lipB* R2 | | CCATGGCGATATCGGATCCGATCTTCAGTGGCGCGGGTA | | None |
| pET-29b F | | CGGATCCGATATCGCCATGG | | None |
| pET-29b R | | AATTCGAGCTCCGTCGACAAGC | | None |
| pBJ113 F | | GGCCGAGCGCCTCGAGCTTCTAGAGTCGACCTGCAGGC | | None |
| pBJ113 R | | TTCCGGGCCGACGTGACGGGATCCCCGGGTACCGAGCT | | None |
| *lipB*-upF | | AGCTCGAGGCGCTCGGCC | | None |
| *lipB*-upR | | CTCCCGGCGGTTCGCAGCCGGTGCCCCGTCACGA | | None |
| *lipB*-downF | | GCTGCGAACCGCCGGGA | | None |
| *lipB*-downR | | CGTCACGTCGGCCCGGAA | | None |
| KG-testF | | GGTGCCAGTGCGGGAGTTTCG | | None |
| KG-testR | | CGAAATGACCGACCAAGC | | None |
| *lipB* F3 | | GGGGTACCATGATTTTTTCGGGCGCGG | | KpnI |
| *lipB* R3 | | GGAATTCTCATGCCGATTCGGTGGCG | | EcoRI |
|  |  | |  |  |
