# Supplementary material for: Genome-wide analysis of lipolytic enzymes and characterization of a high-tolerant carboxylesterase from Sorangium cellulosum
Source: Front Microbiol. 2023 Dec 4;14:1304233. doi: 10.3389/fmicb.2023.1304233 (PMC10725956; doi:10.3389/fmicb.2023.1304233)
Supplement: Supplementary file 3 [file Table_3.DOCX]

| Strain | GenBank | Size (Mb) | Level | PSI-BLAST^a^ | FIMO^b^ |
| --- | --- | --- | --- | --- | --- |
| So ce 56 | GCA_000067165.1 | 13.03 | Complete | 79 | 31 |
| So ce26 | GCA_002950945.1 | 14.56 | Complete | 106 | 44 |
| So ce836 | GCA_004135755.1 | 14.60 | Complete | 99 | 38 |
| So ceGT47 | GCA_004135735.1 | 11.26 | Complete | 62 | 22 |
| So0157-2 | GCA_000418325.1 | 14.78 | Complete | 95 | 34 |
| So0003-19-2 | GCA_001589275.1 | 11.58 | Scaffold | 76 | 29 |
| So0008-312 | GCA_001589285.1 | 11.86 | Scaffold | 80 | 34 |
| So0011-07 | GCA_001589185.1 | 11.99 | Scaffold | 78 | 31 |
| So0149 | GCA_001589205.1 | 11.64 | Scaffold | 84 | 27 |
| So0157-18 | GCA_001589195.1 | 10.91 | Scaffold | 72 | 22 |
| So0157-25 | GCA_001589265.1 | 9.814 | Scaffold | 59 | 26 |
| So0007-03 | GCA_001589215.1 | 14.14 | Contig | 94 | 33 |
| So0163 | GCA_001589295.1 | 13.74 | Contig | 100 | 35 |

**Table S3.** Genome information and identified lipolytic enzymes of 13 *S. cellulosum* strains.

a. Number of proteins retrieved by PSI-BLAST.

b. Number of proteins confirmed by FIMO.
