# Supplementary material for: Genome-wide analysis of lipolytic enzymes and characterization of a high-tolerant carboxylesterase from Sorangium cellulosum
Source: Front Microbiol. 2023 Dec 4;14:1304233. doi: 10.3389/fmicb.2023.1304233 (PMC10725956; doi:10.3389/fmicb.2023.1304233)
Supplement: Supplementary file 4 [file Table_4.DOCX]

| Family | Query sequence | Consensus motif |
| --- | --- | --- |
| I | EIW29778.1 | GXSXG or AXSXG |
| II | AKR05135.1 | GDSL or SGNH |
| III | BAK48590.1 | GXSMG |
| IV | QNC43959.1 | GDSAG and HGG |
| V | AAD07785.1 | GHSFG or GDSAG |
| VI | AYO56657.1 | GXSXG |
| VII | Q01470.1 | GXSAG or GXSGG |
| VIII | AAF59826.1 | SXXK and YXX |
| IX | AAK07742.1 | GYSLG |
| X | WP_004083104.1 | GHSLG or GYSGG |
| XI | ABY60416.1 | GHSLG or GHSMG |
| XII | ACB11220.1 | GHSLGG |
| XIII | AUV46830.1 | GXSLGG |
| XIV | WP_011026365.1 | CHSMG |
| XV | AEN92268.1 | GXSMG or GXSTG |
| XVI | AGF29555.2 | GXSYGXG |
| XVII | ANA76126.1 | GXSQG |
| XVIII | WP_016838941.1 | GVSLG |
| XIX | WP_003975294.1 | GHSAG or GHSXGG |

**Table S4.** Query sequence and consensus motif of each lipolytic enzyme family.
