# Supplementary material for: Genome-wide analysis of lipolytic enzymes and characterization of a high-tolerant carboxylesterase from Sorangium cellulosum
Source: Front Microbiol. 2023 Dec 4;14:1304233. doi: 10.3389/fmicb.2023.1304233 (PMC10725956; doi:10.3389/fmicb.2023.1304233)
Supplement: Supplementary file 5 [file Table_5.DOCX]

**Table S5.** DNA sequence of original and codon optimized *lipB*.

| **Name** | **Sequence** | |  |
| --- | --- | --- | --- |
| Original *lipB* | | ATGATTTTTTCGGGCGCGGGCAGTCAGTGCTCTCGGCAGGGGCAGGCGCCCCCGCTCGGAGAGGCATCGACGGTCATGAGAAGGCGGGAGTTTATGGGAGCTTTGATCAGCGTGGCGGCGCCGGGGTGCGCGTTGGGCGGGGCCGAGGAGGAGGGGCAGCCAGGGCAGGACGCGGGGGCCGGCGCGCTGGCGCCCGCGCGCGAGGTGATGGCGGCGGAGGTGGCGGCGGGGCAGATGCCGGGAGCGGTCTGGCTTGTCGCGCGCGGCGACGACGTGCACGTGGACGCGGTGGGCGTGACCGAGCTCGGCGGCAGCGCGCCCATGCGGCGGGACACGATCTTCCGGATCGCCTCGATGACGAAGGCGGTCACCGCGACGGCGGTGATGATGCTCGTCGAGGAAGGCAAGCTCGACCTCGACGCCCCCGTCGACCGATGGTTGCCGGAGCTCGCGAACCGGAAGGTGCTGGCGCGCATCGATGGCCCGATCGACGAGACGGTGCCCGCGGAGCGGCCGATCACGGTGCGCGACCTGATGACGTTCACGATGGGCTTTGGAATCTCGTTCGACGCATCGTCGCCGATCCAGCGGGCCATCGACGAGCTCGGGCTCGTCAACGCGCAGCCGGTGCCGATGACGCCGCACGGGCCAGACGAGTGGATCCGGCGGCTCGGGACGCTGCCGCTCATGCATCAACCGGGGGCGCAGTGGATGTACAACACGGGGAGCCTGGTGCAGGGGGTGCTCGTGGGGCGCGTGGCCGACCAGGGCTTCGACGCGTTCGTGCGCGAGCGCATCCTCGCGCCGCTCGGGATGCGGGACACCGAATTTCACGTCCCGGCGGACAAGCTCGCTCGGTTCGCGGGCTGCGGTTACTTCACCGACGAGCAGACGGGGGAGAAGACGCGCATGGATCGCGACGGGGCCGAGAGCGCCTATGCCAGCCCGCCGGCGTTCCCTTCGGGCGCCGCCGGGCTCGTGTCGACCGTGGACGACTACCTCCTGTTCGCGCGCATGCTCATGAACGGCGGCGTGCACGAGGGCCGGCGGCTCTTGAGCGCCGCCTCGGTGCGCGAGATGACGGCCGATCACCTCACGCCGGCCCAGAAGGCGGCGTCCTCGTTTTTCCCGGGCTTCTTCGAGACGCACGGCTGGGGTTACGGGCTGGCCGTCATCACGGCGCCGGACGTCGTCTCGGAGGTGCCCGGGCGCTATGGCTGGGACGGCGGGTTCGGGACGTCCTGGATCAATGATCCGGGCCGCGAGCTGATCGGGATCGTGATGACACAATCCGCCGGCTTCCTGTTCTCGGGGGCTCTCGAGCGTTTCTGGCGCAGCGTCTACGTCGCCACCGAATCGGCATGA | |
| Codon optimized *lipB* | | ATCTTCAGTGGCGCGGGTAGTCAGTGCAGTCGTCAAGGTCAAGCCCCACCACTCGGCGAAGCGAGTACGGTGATGCGCCGTCGCGAATTCATGGGCGCGCTCATTAGCGTTGCGGCGCCGGGCTGTGCCCTCGGTGGCGCCGAGGAAGAAGGTCAGCCGGGCCAAGATGCCGGTGCGGGTGCGCTGGCCCCAGCGCGTGAAGTTATGGCCGCGGAAGTTGCGGCCGGTCAGATGCCGGGTGCGGTTTGGCTGGTTGCCCGTGGCGATGACGTTCACGTTGATGCCGTGGGTGTGACCGAACTGGGTGGTAGTGCGCCAATGCGCCGTGACACCATCTTCCGCATCGCCAGCATGACCAAAGCGGTTACCGCCACCGCCGTGATGATGCTGGTTGAGGAGGGCAAACTGGATCTGGATGCGCCGGTTGATCGCTGGCTGCCGGAACTCGCCAACCGCAAAGTTCTGGCCCGTATTGATGGCCCGATTGACGAAACGGTGCCAGCGGAACGCCCGATCACCGTTCGCGATCTGATGACCTTCACCATGGGTTTCGGCATCAGCTTTGATGCCAGCAGTCCGATCCAGCGCGCCATCGACGAACTGGGTCTGGTTAACGCCCAACCGGTGCCGATGACCCCACATGGCCCAGATGAATGGATCCGCCGTCTGGGTACGCTGCCGCTCATGCATCAGCCGGGCGCCCAGTGGATGTACAACACGGGCAGCCTCGTTCAAGGCGTGCTGGTTGGCCGTGTTGCGGACCAAGGCTTTGATGCCTTCGTTCGCGAACGTATTCTGGCCCCGCTCGGTATGCGTGACACGGAATTCCATGTTCCGGCCGATAAACTGGCCCGCTTTGCCGGCTGTGGCTATTTTACGGATGAGCAGACCGGCGAGAAGACCCGTATGGATCGTGACGGTGCCGAAAGCGCCTATGCCAGTCCGCCAGCGTTCCCAAGTGGTGCCGCGGGTCTCGTTAGCACGGTTGATGACTATCTGCTGTTCGCGCGCATGCTGATGAATGGCGGCGTTCACGAAGGCCGTCGTCTGCTGAGTGCGGCGAGCGTTCGCGAAATGACCGCGGATCATCTGACCCCAGCGCAGAAAGCGGCCAGCAGTTTCTTTCCGGGCTTCTTCGAAACCCATGGTTGGGGCTACGGTCTGGCCGTTATCACGGCGCCAGATGTGGTTAGTGAAGTTCCGGGTCGCTACGGTTGGGATGGTGGCTTCGGTACCAGCTGGATCAATGATCCGGGTCGCGAGCTCATCGGTATTGTTATGACGCAGAGCGCGGGTTTTCTGTTTAGCGGCGCGCTGGAACGCTTTTGGCGTAGCGTTTATGTGGCGACGGAAAGCGCC | |
|  |  |  |  |
