# Supplementary material for: Genome-wide analysis of lipolytic enzymes and characterization of a high-tolerant carboxylesterase from Sorangium cellulosum
Source: Front Microbiol. 2023 Dec 4;14:1304233. doi: 10.3389/fmicb.2023.1304233 (PMC10725956; doi:10.3389/fmicb.2023.1304233)
Supplement: Supplementary file 8 [file Table_8.DOCX]

**Table S8.** Classification of 61 repetitive lipolytic enzymes by BLASTP.

BLASTP of lipolytic enzymes repetitive in family III and VI

| Accession | Query Sequence | Query Cover | Identity | E-value | Family |
| --- | --- | --- | --- | --- | --- |
| KYF49770.1 | BAK48590.1 | 5% | 62.50% | 1.5 | VI |
| KYF49770.1 | AYO56657.1 | 91% | 25.52% | 0.032 |  |
| KYF63510.1 | BAK48590.1 | 5% | 62.50% | 1.4 | VI |
| KYF63510.1 | AYO56657.1 | 9% | 52.63% | 0.19 |  |

BLASTP of lipolytic enzymes repetitive in family III and XV

| Accession | Query Sequence | Query Cover | Identity | E-value | Family |
| --- | --- | --- | --- | --- | --- |
| AUX42858.1 | BAK48590.1 | 30% | 29.90% | 3e-04 | XV |
| AUX42858.1 | AEN92268.1 | 32% | 28.05% | 0.12 |  |
| KYF52741.1 | BAK48590.1 | 30% | 31.34% | 0.002 | XV |
| KYF52741.1 | AEN92268.1 | 39% | 26.92% | 0.035 |  |
| KYF65789.1 | BAK48590.1 | 30% | 31.34% | 0.002 | XV |
| KYF65789.1 | AEN92268.1 | 39% | 26.21% | 0.021 |  |
| KYF91208.1 | BAK48590.1 | 30% | 31.34% | 0.002 | XV |
| KYF91208.1 | AEN92268.1 | 39% | 26.92% | 0.020 |  |
| KYF61444.1 | BAK48590.1 | 43% | 26.09% | 0.002 | XV |
| KYF61444.1 | AEN92268.1 | 39% | 25.96% | 0.018 |  |
| KYF73261.1 | BAK48590.1 | 4% | 66.67% | 0.45 | XV |
| KYF73261.1 | AEN92268.1 | 20% | 34.62% | 0.003 |  |
| KYF88728.1 | BAK48590.1 | 17% | 28.07% | 0.31 | XV |
| KYF88728.1 | AEN92268.1 | 71% | 23.20% | 6e-04 |  |
| KYF64822.1 | BAK48590.1 | 31% | 25.26% | 0.23 | XV |
| KYF64822.1 | AEN92268.1 | 14% | 37.84% | 0.003 |  |
| KYG00289.1 | BAK48590.1 | 17% | 28.07% | 0.31 | XV |
| KYG00289.1 | AEN92268.1 | 71% | 23.20% | 6e-04 |  |
| AUX46906.1 | BAK48590.1 | 4% | 66.67% | 0.38 | XV |
| AUX46906.1 | AEN92268.1 | 14% | 37.84% | 0.003 |  |
| CAN97662.1 | BAK48590.1 | 31% | 24.75% | 0.10 | XV |
| CAN97662.1 | AEN92268.1 | 15% | 38.46% | 0.002 |  |
| KYF70022.1 | BAK48590.1 | 17% | 28.07% | 0.067 | XV |
| KYF70022.1 | AEN92268.1 | 76% | 22.01% | 0.001 |  |
| KYG03780.1 | BAK48590.1 | 31% | 25.26% | 0.084 | XV |
| KYG03780.1 | AEN92268.1 | 14% | 37.84% | 0.003 |  |
| AUX34729.1 | BAK48590.1 | 17% | 32.14% | 0.11 | XV |
| AUX34729.1 | AEN92268.1 | 14% | 35.14% | 0.008 |  |
| AUX24155.1 | BAK48590.1 | 29% | 28.89% | 8e-04 | XV |
| AUX24155.1 | AEN92268.1 | 37% | 35.05% | 1e-07 |  |
| AUX22854.1 | BAK48590.1 | 31% | 21.78% | 0.20 | XV |
| AUX22854.1 | AEN92268.1 | 14% | 37.84% | 0.003 |  |
| KYF88659.1 | BAK48590.1 | 8% | 40.00% | 0.24 | XV |
| KYF88659.1 | AEN92268.1 | 14% | 35.14% | 0.007 |  |
| KYF81129.1 | BAK48590.1 | 8% | 42.31% | 0.027 | XV |
| KYF81129.1 | AEN92268.1 | 71% | 25.93% | 0.10 |  |
| KYF81315.1 | BAK48590.1 | 17% | 26.92% | 0.17 | XV |
| KYF81315.1 | AEN92268.1 | 5% | 64.29% | 0.65 |  |

BLASTP of lipolytic enzymes repetitive in family III and XIX

| Accession | Query Sequence | Query Cover | Identity | E-value | Family |
| --- | --- | --- | --- | --- | --- |
| AGP38724.1 | BAK48590.1 | 46% | 27.37% | 6e-09 | III |
| AGP38724.1 | WP_003975294.1 | 40% | 25.98% | 4e-04 |  |
| KYF88005.1 | BAK48590.1 | 46% | 26.84% | 6e-10 | III |
| KYF88005.1 | WP_003975294.1 | 44% | 26.62% | 9e-05 |  |
| AUX27499.1 | BAK48590.1 | 46% | 26.70% | 2e-08 | III |
| AUX27499.1 | WP_003975294.1 | 44% | 27.86% | 1e-04 |  |
| KYF91792.1 | BAK48590.1 | 46% | 26.84% | 6e-10 | III |
| KYF91792.1 | WP_003975294.1 | 44% | 26.62% | 9e-05 |  |
| AUX46554.1 | BAK48590.1 | 46% | 26.98% | 9e-10 | III |
| AUX46554.1 | WP_003975294.1 | 40% | 26.77% | 3e-04 |  |
| KYF72925.1 | BAK48590.1 | 50% | 28.71% | 9e-11 | III |
| KYF72925.1 | WP_003975294.1 | 44% | 25.55% | 2e-04 |  |
| KYF67577.1 | BAK48590.1 | 49% | 25.63% | 3e-08 | III |
| KYF67577.1 | WP_003975294.1 | 44% | 27.89% | 1e-06 |  |

BLASTP of lipolytic enzymes repetitive in family III, XV and XIX

| Accession | Query Sequence | Query Cover | Identity | E-value | Family |
| --- | --- | --- | --- | --- | --- |
| AGP38004.1 | BAK48590.1 | 55% | 24.74% | 6e-11 | III |
| AGP38004.1 | AEN92268.1 | 78% | 26.44% | 1e-06 |  |
| AGP38004.1 | WP_003975294.1 | 51% | 30.00% | 0.16 |  |
| KYG06213.1 | BAK48590.1 | 30% | 32.73% | 6e-10 | III |
| KYG06213.1 | AEN92268.1 | 38% | 31.48% | 6e-06 |  |
| KYG06213.1 | WP_003975294.1 | 72% | 30.00% | 0.11 |  |
| AUX33883.1 | BAK48590.1 | 30% | 31.82% | 1e-09 | III |
| AUX33883.1 | AEN92268.1 | 38% | 31.58% | 1e-06 |  |
| AUX33883.1 | WP_003975294.1 | 51% | 30.00% | 0.015 |  |
| KYF78474.1 | BAK48590.1 | 52% | 25.41% | 2e-11 | III |
| KYF78474.1 | AEN92268.1 | 35% | 30.69% | 5e-07 |  |
| KYF78474.1 | WP_003975294.1 | 36% | 28.93% | 0.001 |  |
| KYF66961.1 | BAK48590.1 | 52% | 23.78% | 3e-09 | III |
| KYF66961.1 | AEN92268.1 | 35% | 29.70% | 4e-06 |  |
| KYF66961.1 | WP_003975294.1 | 36% | 29.75% | 4e-05 |  |
| KYF55858.1 | BAK48590.1 | 41% | 28.76% | 1e-10 | III |
| KYF55858.1 | AEN92268.1 | 75% | 22.89% | 5e-07 |  |
| KYF55858.1 | WP_003975294.1 | 48% | 26.54% | 0.004 |  |
| CAN91965.1 | BAK48590.1 | 54% | 24.48% | 2e-10 | III |
| CAN91965.1 | AEN92268.1 | 38% | 35.65% | 1e-09 |  |
| CAN91965.1 | WP_003975294.1 | 6% | 42.86% | 0.52 |  |
| KYG01755.1 | BAK48590.1 | 30% | 30.00% | 2e-08 | III |
| KYG01755.1 | AEN92268.1 | 38% | 30.70% | 6e-06 |  |
| KYG01755.1 | WP_003975294.1 | 35% | 30.19% | 0.078 |  |
| KYG01380.1 | BAK48590.1 | 30% | 30.00% | 2e-08 | III |
| KYG01380.1 | AEN92268.1 | 38% | 30.70% | 6e-06 |  |
| KYG01380.1 | WP_003975294.1 | 35% | 30.19% | 0.078 |  |

BLASTP of lipolytic enzymes repetitive in family III, VI, XV and XIX

| Accession | Query Sequence | Query Cover | Identity | E-value | Family |
| --- | --- | --- | --- | --- | --- |
| KYF73318.1 | BAK48590.1 | 56% | 24.84% | 8e-09 | III |
| KYF73318.1 | AYO56657.1 | 34% | 25.00% | 0.012 |  |
| KYF73318.1 | AEN92268.1 | 33% | 31.68% | 7e-08 |  |
| KYF73318.1 | WP_003975294.1 | 40% | 29.69% | 3e-04 |  |

BLASTP of lipolytic enzymes repetitive in family IV and VII

| Accession | Query Sequence | Query Cover | Identity | E-value | Family |
| --- | --- | --- | --- | --- | --- |
| AGP42314.1 | QNC43959.1 | 61% | 28.21% | 5e-21 | IV |
| AGP42314.1 | Q01470.1 | 20% | 29.52% | 0.003 |  |
| KYG08346.1 | QNC43959.1 | 57% | 28.57% | 6e-21 | IV |
| KYG08346.1 | Q01470.1 | 20% | 29.52% | 0.003 |  |
| AUX38494.1 | QNC43959.1 | 65% | 28.23% | 9e-22 | IV |
| AUX38494.1 | Q01470.1 | 24% | 29.52% | 0.003 |  |
| CAN99402.1 | QNC43959.1 | 67% | 28.11% | 7e-22 | IV |
| CAN99402.1 | Q01470.1 | 20% | 30.00% | 7e-04 |  |
| KYF98583.1 | QNC43959.1 | 71% | 27.10% | 7e-21 | IV |
| KYF98583.1 | Q01470.1 | 20% | 29.13% | 0.001 |  |
| KYF94247.1 | QNC43959.1 | 71% | 27.10% | 1e-20 | IV |
| KYF94247.1 | Q01470.1 | 20% | 29.13% | 0.001 |  |
| KYF71002.1 | QNC43959.1 | 57% | 28.57% | 6e-20 | IV |
| KYF71002.1 | Q01470.1 | 20% | 28.28% | 0.005 |  |
| AUX22000.1 | QNC43959.1 | 66% | 37.86% | 5e-40 | IV |
| AUX22000.1 | Q01470.1 | 20% | 32.32% | 3e-12 |  |
| KYF57731.1 | QNC43959.1 | 57% | 28.57% | 3e-21 | IV |
| KYF57731.1 | Q01470.1 | 20% | 29.29% | 0.002 |  |
| AUX49088.1 | QNC43959.1 | 65% | 28.23% | 2e-22 | IV |
| AUX49088.1 | Q01470.1 | 26% | 26.61% | 9e-04 |  |

BLASTP of lipolytic enzymes repetitive in family X and XIII

| Accession | Query Sequence | Query Cover | Identity | E-value | Family |
| --- | --- | --- | --- | --- | --- |
| AGP33472.1 | WP_004083104.1 | 30% | 28.87% | 0.004 | XIII |
| AGP33472.1 | AUV46830.1 | 81% | 27.68% | 6e-04 |  |
| KYF56946.1 | WP_004083104.1 | 30% | 28.87% | 0.004 | XIII |
| KYF56946.1 | AUV46830.1 | 81% | 27.68% | 6e-04 |  |
| AUX19922.1 | WP_004083104.1 | 31% | 28.71% | 0.005 | XIII |
| AUX19922.1 | AUV46830.1 | 47% | 25.78% | 9e-05 |  |
| CAN90466.1 | WP_004083104.1 | 30% | 30.21% | 0.002 | XIII |
| CAN90466.1 | AUV46830.1 | 81% | 20.18% | 0.13 |  |
| KYF78559.1 | WP_004083104.1 | 34% | 27.35% | 0.003 | XIII |
| KYF78559.1 | AUV46830.1 | 81% | 27.68% | 0.001 |  |
| KYF89216.1 | WP_004083104.1 | 22% | 28.87% | 0.001 | XIII |
| KYF89216.1 | AUV46830.1 | 81% | 27.68% | 6e-04 |  |
| KYF55408.1 | WP_004083104.1 | 34% | 27.35% | 0.003 | XIII |
| KYF55408.1 | AUV46830.1 | 81% | 27.68% | 0.001 |  |
| KYG07843.1 | WP_004083104.1 | 30% | 28.87% | 0.003 | XIII |
| KYG07843.1 | AUV46830.1 | 81% | 27.68% | 7e-04 |  |
| KYF64212.1 | WP_004083104.1 | 34% | 28.45% | 0.003 | XIII |
| KYF64212.1 | AUV46830.1 | 80% | 27.93% | 0.002 |  |
| AUX28492.1 | WP_004083104.1 | 34% | 27.35% | 0.003 | XIII |
| AUX28492.1 | AUV46830.1 | 81% | 28.57% | 4e-04 |  |
| KYG01529.1 | WP_004083104.1 | 22% | 28.87% | 0.001 | XIII |
| KYG01529.1 | AUV46830.1 | 81% | 27.68% | 6e-04 |  |
| AUX39195.1 | WP_004083104.1 | 30% | 28.87% | 0.004 | XIII |
| AUX39195.1 | AUV46830.1 | 40% | 26.79% | 0.001 |  |
| KYF71251.1 | WP_004083104.1 | 26% | 27.73% | 0.003 | XIII |
| KYF71251.1 | AUV46830.1 | 40% | 26.79% | 0.004 |  |
