# Supplementary material for: Genome-wide analysis of lipolytic enzymes and characterization of a high-tolerant carboxylesterase from Sorangium cellulosum
Source: Front Microbiol. 2023 Dec 4;14:1304233. doi: 10.3389/fmicb.2023.1304233 (PMC10725956; doi:10.3389/fmicb.2023.1304233)
Supplement: Supplementary file 9 [file Table_9.docx]

**Table S9.** Potential lipolytic enzymes in each *Sorangium cellulosum* strain.

| family I | AGP36238.1 AGP32429.1 AGP39341.1 AGP39712.1 |
| --- | --- |
| family III | AGP38004.1 AGP39165.1 AGP38724.1 |
| family IV | AGP42314.1 |
| family V | AGP36803.1 |
| family VI | AGP33132.1 AGP33369.1 AGP34778.1 AGP35429.1 AGP39686.1 |
| family VII | AGP38017.1 |
| family VIII | AGP34009.1 AGP34771.1 AGP36607.1 AGP37456.1 AGP39702.1  AGP40016.1 AGP40858.1 |
| family X | AGP35394.1 |
| family XIII | AGP32158.1 AGP34741.1 AGP35710.1 AGP33472.1 |
| family XV | AGP33865.1 AGP34164.1 AGP36830.1 AGP38422.1 AGP38789.1 |
| family XIX | AGP33915.1 AGP37715.1 |

So0157-2

So ce26

| family I | AUX41997.1 AUX42800.1 AUX46068.1 AUX47999.1 |
| --- | --- |
| family III | AUX44424.1 AUX46402.1 AUX48485.1 AUX46554.1 |
| family IV | AUX49088.1 |
| family V | AUX42823.1 AUX48302.1 |
| family VI | AUX39854.1 AUX43577.1 AUX43712.1 AUX46039.1 |
| family VII | AUX38922.1 AUX38926.1 AUX39607.1 AUX42370.1 AUX42382.1 |
| family VIII | AUX40628.1 AUX40991.1 AUX42563.1 AUX43432.1 AUX44525.1  AUX46056.1 AUX46100.1 AUX47433.1 |
| family X | AUX42529.1 |
| family XIII | AUX40010.1 AUX40600.1 AUX39195.1 |
| family XV | AUX39827.1 AUX40419.1 AUX42858.1 AUX43387.1 AUX44740.1  AUX46491.1 AUX46906.1 AUX47534.1 AUX48612.1 |
| family XVII | AUX42381.1 |
| family XIX | AUX43940.1 AUX44199.1 |

So ce836

| family I | AUX31693.1 AUX32297.1 AUX35375.1 AUX35664.1 |
| --- | --- |
| family III | AUX33883.1 AUX33915.1 |
| family IV | AUX38494.1 |
| family V | AUX32330.1 AUX34817.1 |
| family VI | AUX28066.1 AUX33044.1 AUX33270.1 AUX34673.1 AUX35634.1  AUX36999.1 |
| family VII | AUX30833.1 AUX33896.1 |
| family VIII | AUX29081.1 AUX29304.1 AUX29926.1 AUX32123.1 AUX33117.1  AUX35652.1 AUX35993.1 AUX36898.1 AUX37028.1 |
| family X | AUX30678.1 |
| family XIII | AUX29410.1 AUX29888.1 AUX33057.1 AUX36768.1 AUX28492.1 |
| family XV | AUX28915.1 AUX29302.1 AUX32359.1 AUX34371.1 AUX34729.1 |
| family XIX | AUX33534.1 |

So ceGT47

| family I | AUX22192.1 AUX22664.1 AUX25114.1 AUX25830.1 |
| --- | --- |
| family III | AUX27499.1 |
| family IV | AUX22000.1 |
| family V | AUX22671.1 AUX26939.1 |
| family VI | AUX21266.1 |
| family VIII | AUX20903.1 AUX25103.1 AUX26225.1 AUX26392.1 AUX26401.1 |
| family XIII | AUX20843.1 AUX21728.1 AUX19922.1 |
| family XV | AUX20440.1 AUX21662.1 AUX22854.1 AUX24155.1 AUX27322.1 |

So ce 56

| family I | CAN91061.1 CAN93141.1 CAN93630.1 CAN96433.1 |
| --- | --- |
| family III | CAN91669.1 CAN91965.1 |
| family IV | CAN99402.1 |
| family V | CAN93673.1 CAN98287.1 |
| family VI | CAN90905.1 CAN91349.1 CAN93435.1 CAN94263.1 CAN94347.1  CAN95184.1 CAN96541.1 |
| family VII | CAN94872.1 |
| family VIII | CAN91653.1 CAN96421.1 CAN96810.1 CAN97941.1 |
| family XIII | CAN91168.1 CAN91616.1 CAN92652.1 CAN90466.1 |
| family XV | CAN90851.1 CAN91055.1 CAN93635.1 CAN95173.1 CAN97662.1 |
| family XIX | CAN94526.1 |

So0003-19-2

| family I | KYF82584.1 KYF80891.1 KYF75406.1 KYF64409.1 |
| --- | --- |
| family III | KYF78474.1 KYF63617.1 |
| family V | KYF78200.1 KYF64673.1 KYF64242.1 |
| family VI | KYF76792.1 KYF70490.1 KYF69724.1 KYF63510.1 |
| family VII | KYF84066.1 |
| family VIII | KYF80403.1 KYF77404.1 KYF74936.1 KYF74472.1 |
| family XIII | KYF79317.1 KYF61247.1 KYF64212.1 |
| family XV | KYF81315.1 KYF81129.1 KYF80462.1 KYF78079.1 KYF73261.1  KYF69209.1 KYF65789.1 |
| family XIX | KYF83533.1 |

So0008-312

| family I | KYF74439.1 KYF73533.1 KYF72199.1 KYF69411.1 |
| --- | --- |
| family III | KYF66961.1 KYF72925.1 KYF67577.1 |
| family IV | KYF71002.1 KYF67701.1 |
| family V | KYF72020.1 KYF70133.1 KYF67348.1 |
| family VI | KYF73581.1 KYF72352.1 KYF67190.1 KYF67026.1 KYF65311.1  KYF63411.1 |
| family VII | KYF72609.1 KYF65484.1 |
| family VIII | KYF66633.1 KYF65501.1 KYF64180.1 KYF64166.1 KYF63027.1 |
| family XIII | KYF64559.1 KYF71251.1 |
| family XV | KYF72381.1 KYF70022.1 KYF65101.1 KYF63765.1 KYF61444.1 |
| family XVII | KYF65485.1 |
| family XIX | KYF69646.1 |

So0157-18

| family I | KYF65015.1 KYF56153.1 KYF55644.1 KYF51795.1 |
| --- | --- |
| family III | KYF47165.1 |
| family VI | KYF57682.1 KYF56659.1 KYF55636.1 |
| family VII | KYF59491.1 |
| family VIII | KYF56385.1 KYF56132.1 KYF55679.1 KYF50355.1 KYF49674.1 |
| family X | KYF57174.1 |
| family XIII | KYF52599.1 KYF56946.1 |
| family XV | KYF64822.1 KYF53583.1 KYF53309.1 |
| family XIX | KYF59845.1 KYF51940.1 |

So0157-25

| family I | KYF59747.1 KYF57889.1 KYF53961.1 |
| --- | --- |
| family III | KYF55858.1 KYF52412.1 |
| family IV | KYF57731.1 |
| family V | KYF55307.1 KYF51635.1 |
| family VI | KYF53518.1 KYF49770.1 |
| family VII | KYF52609.1 |
| family VIII | KYF60066.1 KYF56707.1 KYF56047.1 KYF55935.1 |
| family XIII | KYF58868.1 KYF48076.1 KYF55408.1 |
| family XV | KYF59853.1 KYF59640.1 KYF59039.1 KYF56559.1 KYF55680.1  KYF53557.1 KYF52741.1 |
| family XIX | KYF52109.1 |

So0149

| family I | KYG02267.1 KYF96539.1 KYF84122.1 KYF76634.1 |
| --- | --- |
| family III | KYG01380.1 KYF83863.1 KYF91792.1 |
| family IV | KYF98583.1 |
| family V | KYF98711.1 KYF92055.1 |
| family VI | KYF96492.1 KYF92807.1 KYF87995.1 |
| family VII | KYG02350.1 |
| family VIII | KYG01959.1 KYF88391.1 KYF86526.1 KYF75151.1 |
| family XIII | KYG01000.1 KYF91685.1 KYG01529.1 |
| family XV | KYG00289.1 KYF98952.1 KYF81361.1 KYF77331.1 |
| family XVII | KYG02010.1 |
| family XIX | KYF79271.1 |

So0011-07

| family I | KYF96846.1 KYF86412.1 KYF85177.1 KYF84980.1 KYF73215.1 |
| --- | --- |
| family III | KYF85756.1 KYF73318.1 |
| family V | KYF92013.1 KYF83523.1 |
| family VI | KYF94987.1 KYF89812.1 KYF89148.1 KYF80126.1 KYF76862.1 |
| family VII | KYF83295.1 |
| family VIII | KYF87719.1 KYF84167.1 KYF76898.1 KYF74867.1 KYF74524.1  KYF74076.1 |
| family XIII | KYF85839.1 KYF80721.1 KYF78559.1 |
| family XV | KYF97205.1 KYF91208.1 KYF88659.1 KYF84281.1 KYF83420.1  KYF72866.1 |
| family XIX | KYF74161.1 |

So0007-03

| family I | KYG11340.1 KYG10939.1 KYG05166.1 KYG04624.1 |
| --- | --- |
| family III | KYG06213.1 KYG00781.1 |
| family IV | KYG08346.1 |
| family V | KYG04573.1 |
| family VI | KYG10967.1 KYG08140.1 KYG06617.1 |
| family VII | KYG05894.1 KYG05131.1 |
| family VIII | KYG10588.1 KYG09782.1 KYG09641.1 KYG07360.1 KYG06619.1  KYG04789.1 KYG03793.1 |
| family X | KYG06003.1 |
| family XIII | KYG07049.1 KYG06656.1 KYG05696.1 KYG07843.1 |
| family XV | KYG07488.1 KYG07415.1 KYG07210.1 KYG04548.1 KYG03780.1 |
| family XVII | KYG05896.1 |
| family XIX | KYG03450.1 KYG01823.1 |

So0163

| family I | KYG02355.1 KYG00585.1 KYF99698.1 KYF93828.1 |
| --- | --- |
| family III | KYG01755.1 KYF99611.1 KYF88005.1 |
| family IV | KYF94247.1 |
| family V | KYF97038.1 KYF96107.1 |
| family VI | KYF96983.1 KYF94963.1 KYF88833.1 KYF88504.1 |
| family VII | KYF95152.1 KYF87270.1 |
| family VIII | KYF98097.1 KYF97629.1 KYF96915.1 KYF94060.1 KYF91733.1  KYF88190.1 |
| family XIII | KYF99785.1 KYF92180.1 KYF85948.1 KYF89216.1 |
| family XV | KYG00394.1 KYF99566.1 KYF98060.1 KYF94637.1 KYF93292.1  KYF88728.1 |
| family XVII | KYF87272.1 |
| family XIX | KYF94371.1 KYF93124.1 |
