# Supplementary material for: Genome-wide analysis of lipolytic enzymes and characterization of a high-tolerant carboxylesterase from Sorangium cellulosum
Source: Front Microbiol. 2023 Dec 4;14:1304233. doi: 10.3389/fmicb.2023.1304233 (PMC10725956; doi:10.3389/fmicb.2023.1304233)
Supplement: Supplementary file 10 [file Table_10.DOCX]

**Table S10.** Top 10 identified structurally analogous proteins in PDB by I-TASSER.

| **PDB ID** | **TM-score** | **RMSD^a^** | **IDEN^b^** | **Cov^c^** |
| --- | --- | --- | --- | --- |
| 4p6bA | 0.832 | 0.83 | 0.255 | 0.839 |
| 4iviA | 0.785 | 2.18 | 0.292 | 0.833 |
| 3hlgA | 0.743 | 3.23 | 0.230 | 0.833 |
| 6kjcA | 0.733 | 3.55 | 0.254 | 0.835 |
| 1ci8A | 0.730 | 2.42 | 0.311 | 0.786 |
| 6kjhA | 0.712 | 2.63 | 0.239 | 0.773 |
| 5gkvA | 0.691 | 2.97 | 0.214 | 0.775 |
| 2qmiA | 0.673 | 2.90 | 0.219 | 0.745 |
| 3zytA | 0.668 | 3.18 | 0.162 | 0.762 |
| 4y7pA | 0.667 | 2.90 | 0.217 | 0.740 |

a. RMSD is the RMSD between residues that are structurally aligned by TM-align.

b. IDEN is the percentage sequence identity in the structurally aligned region.

c. Cov represents the coverage of the alignment by TM-align and is equal to the number of structurally aligned residues divided by length of the query protein.
