# Supplementary material for: Genome-wide analysis of lipolytic enzymes and characterization of a high-tolerant carboxylesterase from Sorangium cellulosum
Source: Front Microbiol. 2023 Dec 4;14:1304233. doi: 10.3389/fmicb.2023.1304233 (PMC10725956; doi:10.3389/fmicb.2023.1304233)
Supplement: Supplementary file 11 [file Data_Sheet_1.PDF]

Supplementary Figures

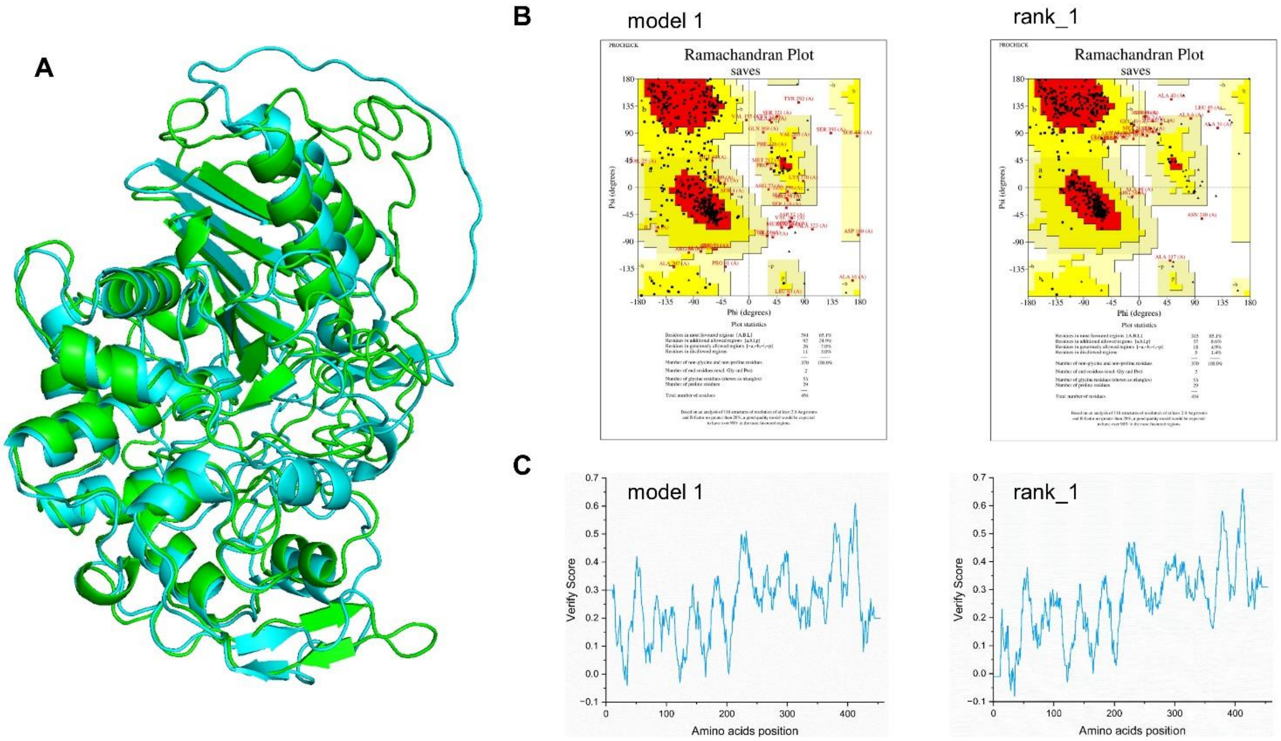

Supplementary Figure 1. Assessment of three-dimensional structures established by I-TASSER and AlphaFold2. (**A**) Alignment of 3D structures, model 1 (C-score: -0.65, TM-Score: 0.63±0.14, RMSD: 8.6±4.5) predicted by I-TASSER which is colored in green and model rank_1 (pLDDT:87.3, pTM:0.857) constructed by AlphaFold2 colored in cyan. (**B**) Ramachandran plot for the predicted model 1 constructed by I-TASSER (left) and rank_1 by AlphaFold2 (right). The plots were created by PROCHECK in SAVES. (**C**) Verify3D scores for each amino acid in model 1 established by I-TASSER (left) and rank_1 by AlphaFold2 (right).

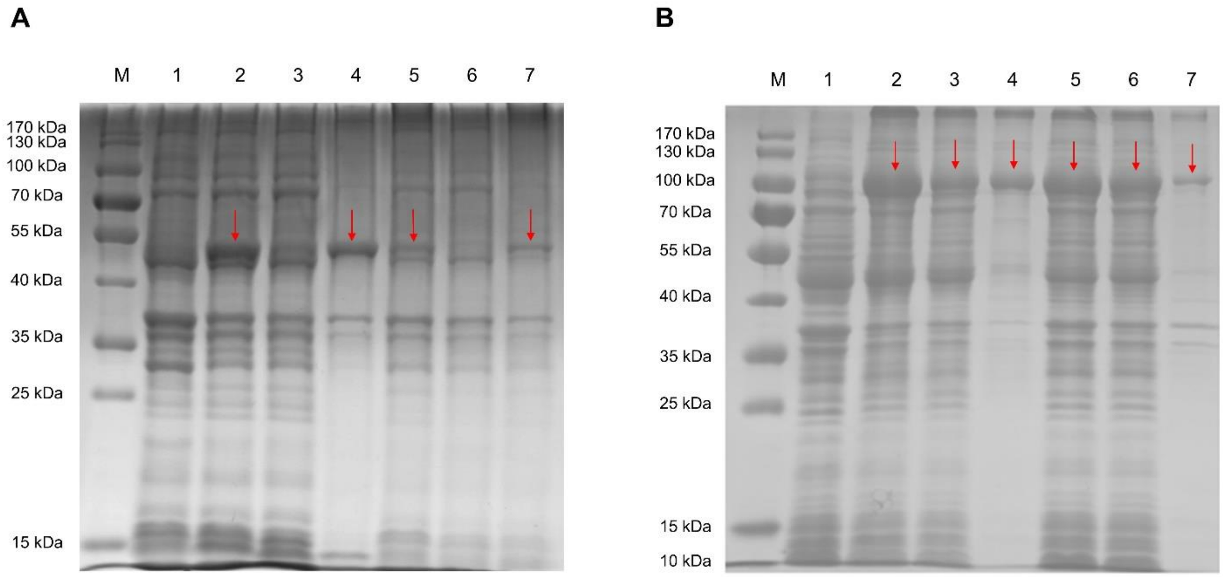

Supplementary Figure 2. Expression of recombinant LipB analyzed by SDS-PAGE. Inducible expression of His-LipB (**A**) and inducible expression of LipB (**B**). Lane M, protein marker; Lane 1, crude protein extracts of recombinant cells without IPTG; Lane 2, crude protein extracts of recombinant cells induced with 1 mM IPTG at 37 °C; Lane 3, supernatant of recombinant cells induced with 1 mM IPTG at 37 °C; Lane 4, sediment of recombinant cells induced with 1 mM IPTG at 37 °C; Lane 5, crude protein extracts of recombinant cells induced with 0.1 mM IPTG at 16 °C; Lane 6, supernatant of recombinant cells induced with 0.1 mM IPTG at 16 °C; Lane 7, sediment of recombinant cells induced with 0.1 mM IPTG at 16 °C. Recombinant proteins are marked by red arrows.

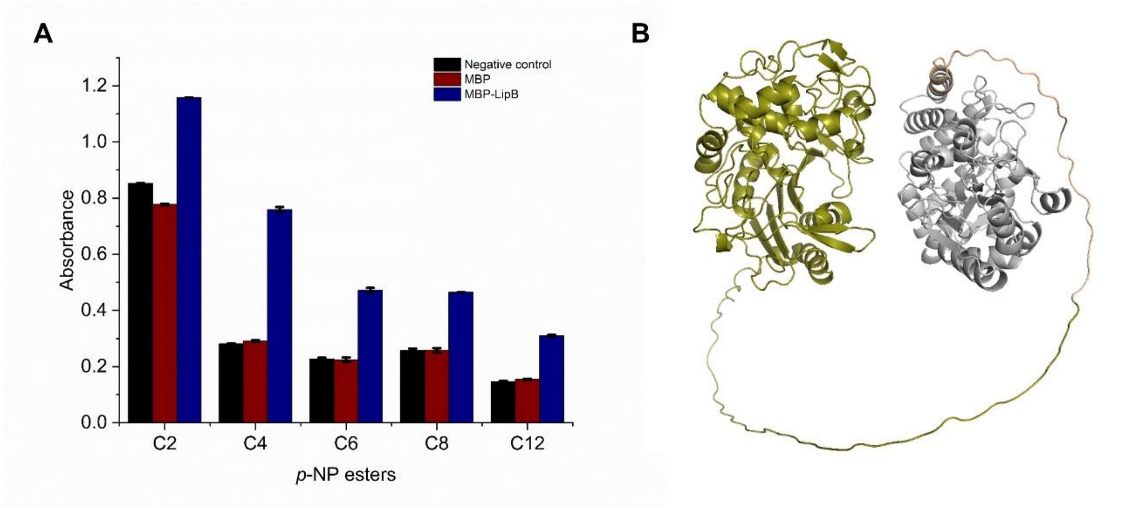

Supplementary Figure 3. (**A**) The absorbance of reaction solution. Negative control: Mixture containing *p*-NP esters only; MBP: Solution of *p*-NP esters incubation with MBP protein; MBP-LipB: Solution of *p*-NP esters incubation with MBP-LipB. *p*-NP acetate (C2), *p*-NP butyrate (C4), *p*-NP hexanoate (C6), *p*-NP octanoate (C8), *p*-NP decanoate (C10) and *p*-NP laurate (C12). (**B**) Three-dimensional structure of recombinant protein MBP-LipB. MBP-tag, LipB and the linker were colored in grey, deep olive and wheat, respectively.

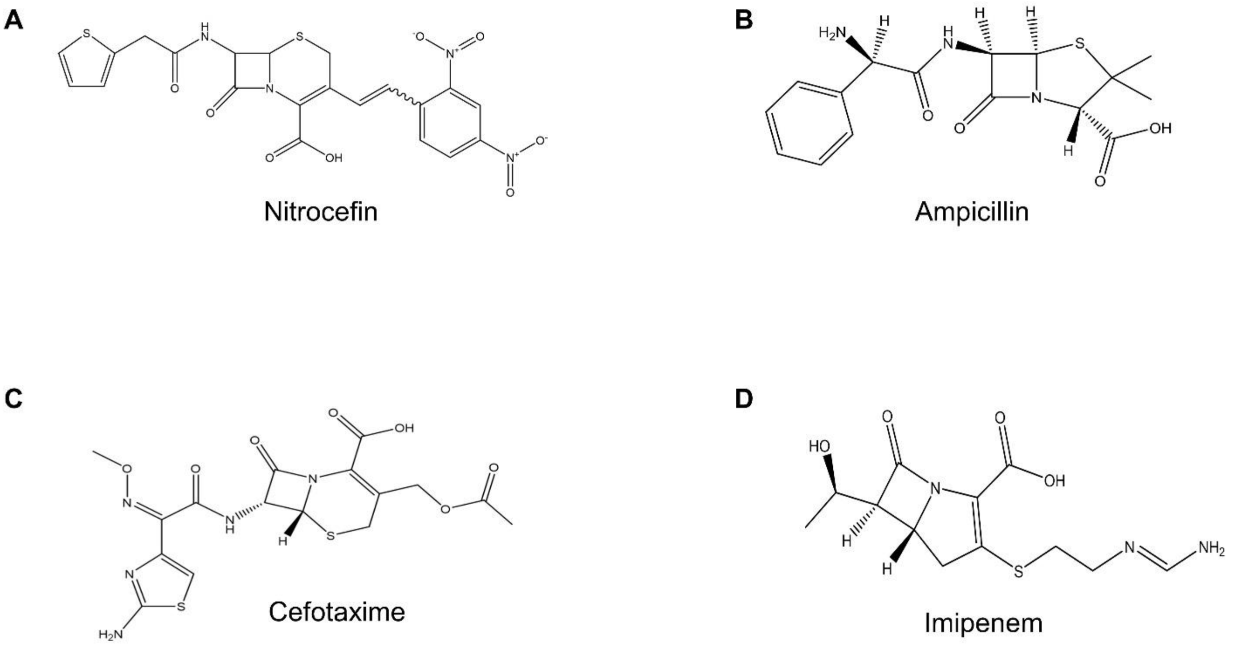

Supplementary Figure 4. Molecular structures of (**A**) nitrocefin, (**B**) ampicillin, (**C**) cefotaxime and (**D**) imipenem.

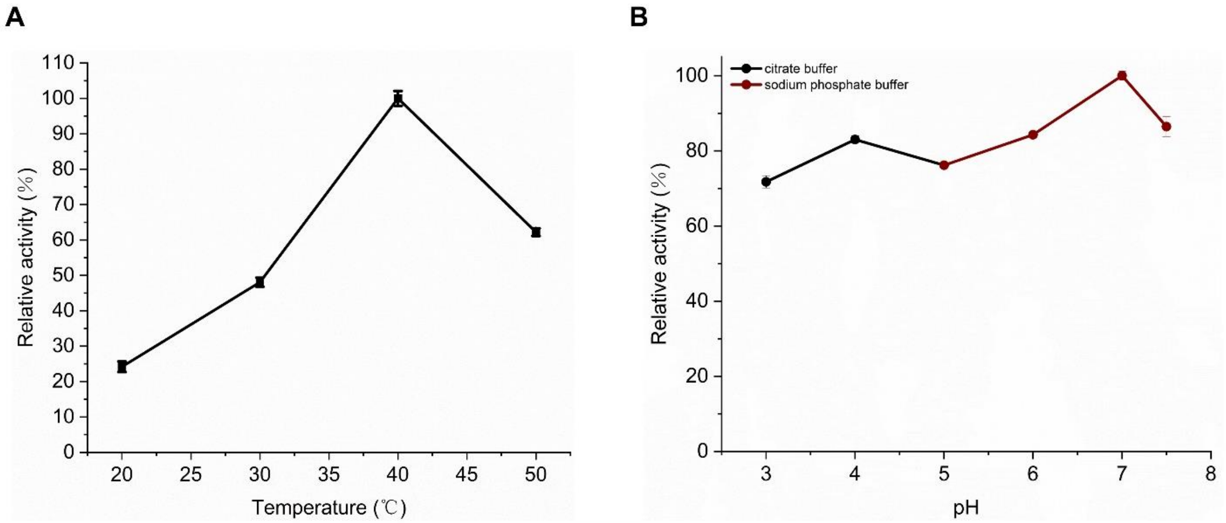

Supplementary Figure 5. (**A**) Effect of temperature on β-lactamase activity of LipB. (**B**) Effect of pH on β-lactamase activity of LipB.

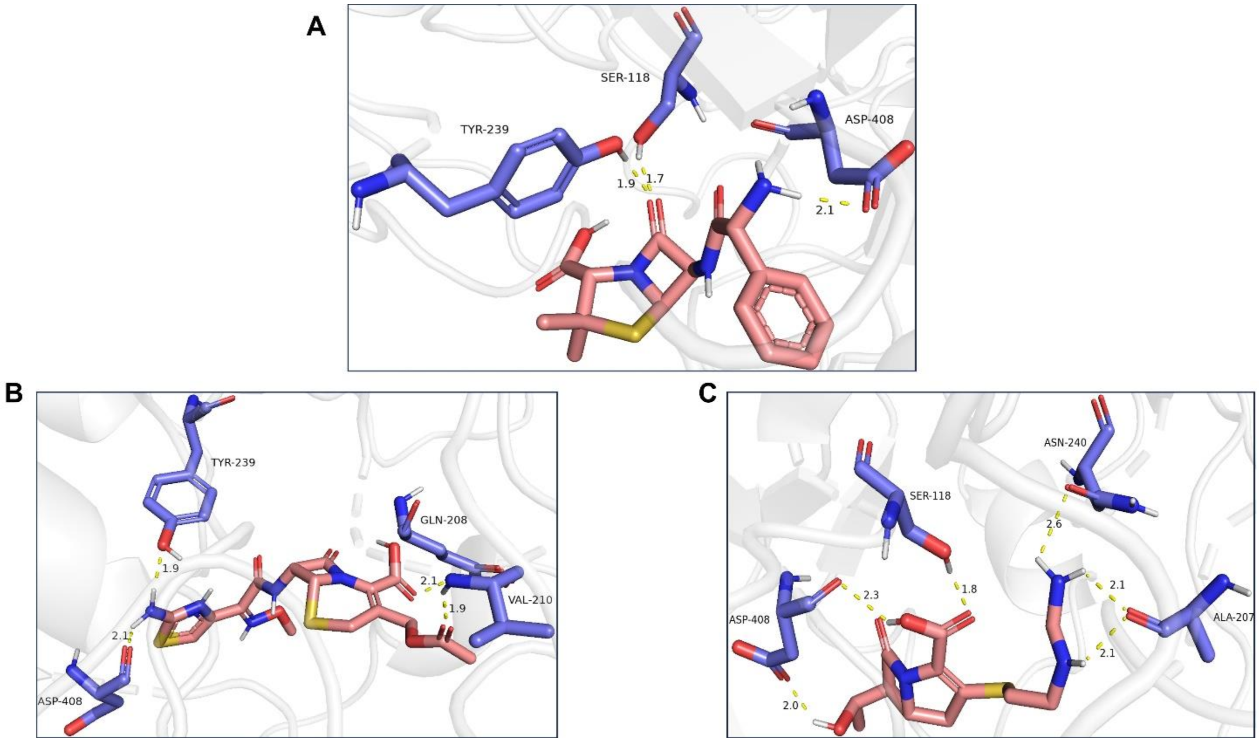

Supplementary Figure 6. The potential hydrogen bond interactions between LipB and poor substrates ampicillin (**A**), cefotaxime (**B**), imipenem (**C**).

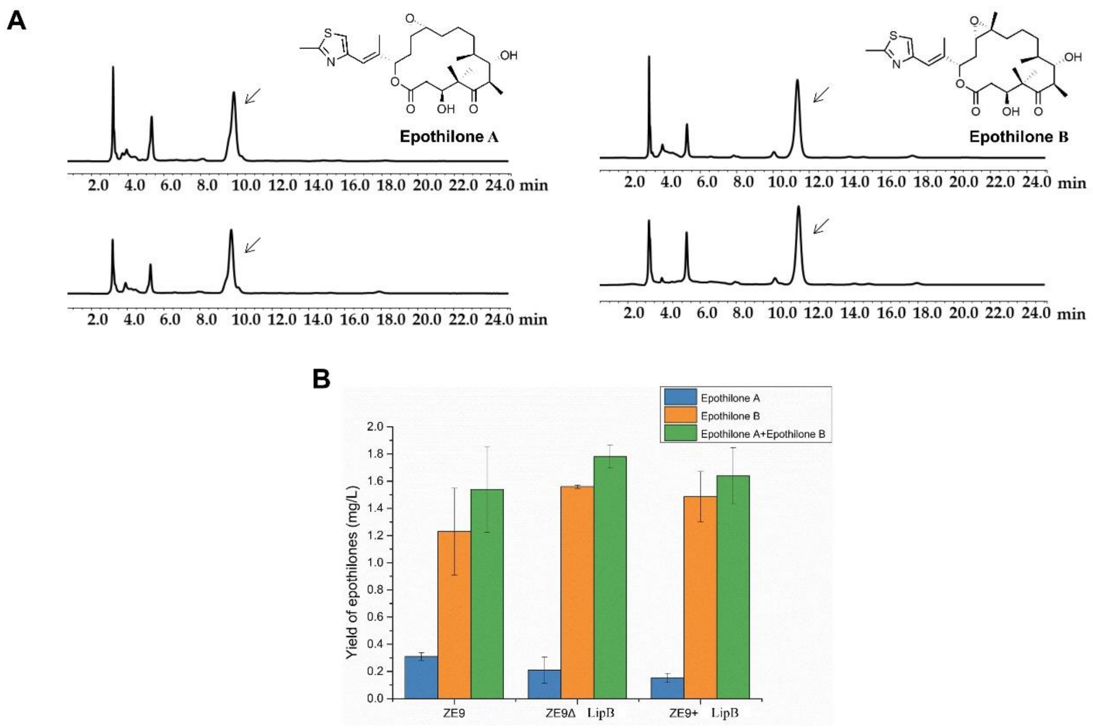

Supplementary Figure 7. Activity assays of LipB on epothilones. (A) HPLC analysis for the production of epothilones treated with (the below pictures) and without (the upper pictures) MBP-LipB. The substrate peaks of epothilone A and epothilone B are at 9.8 min and 11.3 min, marked with arrows. (B) Yields of epothilones A and B in ZE9, ZE9ΔlipB and ZE9+lipB.
